# The Anomalous Diffusion of a Tumor Invading with Different Surrounding Tissues

**DOI:** 10.1371/journal.pone.0109784

**Published:** 2014-10-13

**Authors:** Chongming Jiang, Chunyan Cui, Li Li, Yuanzhi Shao

**Affiliations:** 1 School of Physics and Engineering, Sun Yat-sen University, Guangzhou, China; 2 State Key Laboratory of Oncology in South China, Sun Yat-sen University Cancer Center, Guangzhou, China; 3 Institut Franco-Chinois de L'Énergie Nucléaire, Sun Yat-sen University, Zhuhai, China; University of Michigan School of Medicine, United States of America

## Abstract

We simulated the invasion of a proliferating, diffusing tumor within different surrounding tissue conditions using a hybrid mathematical model. The *in silico* invasion of a tumor was addressed systematically for the first time within the framework of a generalized diffusion theory. Our results reveal that a tumor not only migrates using typical Fickian diffusion, but also migrates more generally using subdiffusion, superdiffusion, and even ballistic diffusion, with increasing mobility of the tumor cell when haptotaxis and chemotaxis toward the host tissue surrounding the proliferative tumor are involved. Five functional terms were included in the hybrid model and their effects on a tumor's invasion were investigated quantitatively: haptotaxis toward the extracellular matrix tissue that is degraded by matrix metalloproteinases; chemotaxis toward nutrients; cell-cell adhesion; the proliferation of the tumor; and the immune response toward the tumor. Haptotaxis and chemotaxis, which are initiated by extracellular matrix and nutrient supply (i.e., glucose) respectively, as well as cell-cell adhesions all drastically affect a tumor's diffusion mode when a tumor invades its surrounding host tissue and proliferates. We verified the *in silico* invasive behavior of a tumor by analyzing experimental data gathered from the *in vitro* culturing of different tumor cells and clinical imaging observations that used the same approach as was used to process the simulation data. The different migration modes of a tumor suggested by the simulations generally conform to the results observed in cell cultures and in clinical imaging. Our study not only discloses some migration modes of a tumor that proliferates and invades under different host tissues conditions, but also provides a heuristic method to characterize the invasion of a tumor in clinical medical imaging analysis.

## Introduction

Tumor invasion is one of the crucial characteristic stages in the evolution of a tumor and is the main clinical sign of a malignant tumor. More than 80% of clinical tumor patients die from tumor invasion and metastasis. Some tumors, such as gliomas, are able to invade at an early stage [1]. Tumor migration has been ascribed to multiple factors, such as the inherent nature of tumor cells themselves and their surrounding host tissue [2]–[4].

The host tissue is usually referred to as the tumor's microenvironment and includes elements such as the extracellular matrix (ECM), the matrix metalloproteinases (MMPs), the nutrient supply surrounding the tumor, and the cell-cell adhesion. The ECM is a complex mixture of macromolecules, such as collagens, laminin, fibronectin, and vitronectin. It may also contain growth factors and may be degraded to release special fragments that promote tumor growth. By definition, haptotaxis is the directed migratory response of cells to gradients of fixed or bound chemicals, i.e. a response to gradients of bound MM such as fibronectin. Some efforts have been done to characterize such directed movement [5]–[7]. The physical removal of the ECM can allow a tumor to spread, and its degradation may produce a positive biological effect on tumor invasion [8]. The implications of the MMPs in tumor invasion are described in the references by Matrisian [9], Mignatti and Rifkin [10], and Thorgeirsson et al. [11]. The MMPs can open migratory pathways and alter cell adhesion properties by regulating several classes of cell-surface receptors, such as cadherins, CD-44, integrins, and receptors for fibronectin, laminin, and vitronectin. These receptors negatively regulate cell motility and growth through cell-cell and cell-matrix interactions [12]–[14]. The proteolytic degradation of the receptors and the ECM components can release tumor cells from these constraints. Aside from practical considerations, it is of academic significance to investigate the invasion of tumors by taking into account both cell-cell and cell-matrix interactions. These studies lay the mathematical foundation for studying the proliferative growth and diffusion of a tumor, which will be addressed specifically below.

Ordinary differential equations (ODEs) have been successfully used to formulate the proliferative growth process of a tumor, such as Gompertzian growth, logistic growth, and exponential growth (see Araujo et al. [15] for a review). Deterministic reaction-diffusion equations have been used to model the spatial spread of a tumor, both at an early stage in its growth [16], [17] and at a later invasive stage [2], [4], [18]–[22]. Sander et al. [23], Anderson et al. [18], and Kim et al. [19] used similar mathematical models to reproduce the numerical patterns of tumor invasion. Their models consisted of some partial differential equations (PDEs) that dealt with the tumor cell density, nutrient concentration, and homotypic factor concentration. Their studies made clear that a very strong homotypic attraction and strong chemotaxis are indispensable for the formation of a branching pattern. Verbeni et al. [24] found that some morphogens, e.g. Hedgehog (Hh) molecules, may not freely diffuse; Wu et al. [25] found that cell migration through 3D ECM does not follow a random walk. They did not, however, address the anomalous diffusion that can occur in a tumor invasion process.

Diffusion is one of the most ubiquitous transport mechanisms in nature. Quantifying diffusion processes remains a critical issue of practical and academic importance in science and engineering fields. The description of diffusion at a macroscopic level is based on Fick's hypothesis [26], [27], which assumes proportionality between the flux and the concentration gradient of a diffusing physical quantity. A direct consequence of Fick's approach is that one-dimensional diffusion along a concentration gradient is expected to scale as *t^0.5^* in homogeneous and isotropic systems. Fickian diffusion has been accepted as universal for more than a hundred years and has been used to account for a variety of phenomena, including heat conduction, moisture transport in porous materials and ionic and membrane transport [28]–[30]. In recent decades, however, diffusion deviating from the expected *t^0.5^* scaling law has been increasingly reported in physics [31], [32] and in chemistry and biology studies [33]–[36], in addition to being been grouped under the general concept of anomalous diffusion [37].

We explored the diffusion features of a tumor that proliferates and invades inside the surrounding tissue with three interactive sub-processes: the tumor cells adhere to the ECM components, the tumor cells secrete MMPs and degrade the ECM and the tumor cells invade the surrounding tissue. Our simulation shows that the diffusion behavior of an invasive tumor, depending on the surrounding matrix, may vary considerably from a typical Fickian diffusion to anomalous diffusion (subdiffusion, superdiffusion, and ballistic diffusion). More specifically, the migration of a tumor inside a complex surrounding matrix is generally characteristic of an anomalous diffusion. Both *in vitro* cell cultures and clinical medical imaging data from tumor invasions support the simulations. The effects of haptotaxis toward the ECM, chemotaxis toward the nutrient supply, cell-cell adhesion, tumor proliferation, and immune regulation on tumor invasive diffusion are discussed in detail.

The remainder of this paper is organized as follows. In Sec. II, we describe the mathematical model and the criteria used to classify diffusion types. In Sec. III, we present the important results of the simulation, *in vitro* cell cultures, and clinical imaging. The discussion and analysis are performed in Sec. IV. The concluding remarks are included in Sec. IV. The paper also includes supporting information in which some simulation results, original clinical images, and *in vitro* cell culture data and the methods used to quantitatively process the data are given.

## Methods

### Ethics Statement

This study did not involve any human experiments as well as treatment processes. The clinical images of tumors were acquired in ordinary medical examinations for patients in Sun Yat-sen University Cancer Center (cancer hospital), these examinations were carried out totally for therapy and no additional drugs or measures were used. This study was approved by the ethics committee of Sun Yat-sen University Cancer Center and every effort was also made to maximize the protection of patients' privacy (e.g. the data were analyzed anonymously). Both the research materials and results are used for scientific purposes without conflict of interests.

### Mathematical model

**Figure 1:**
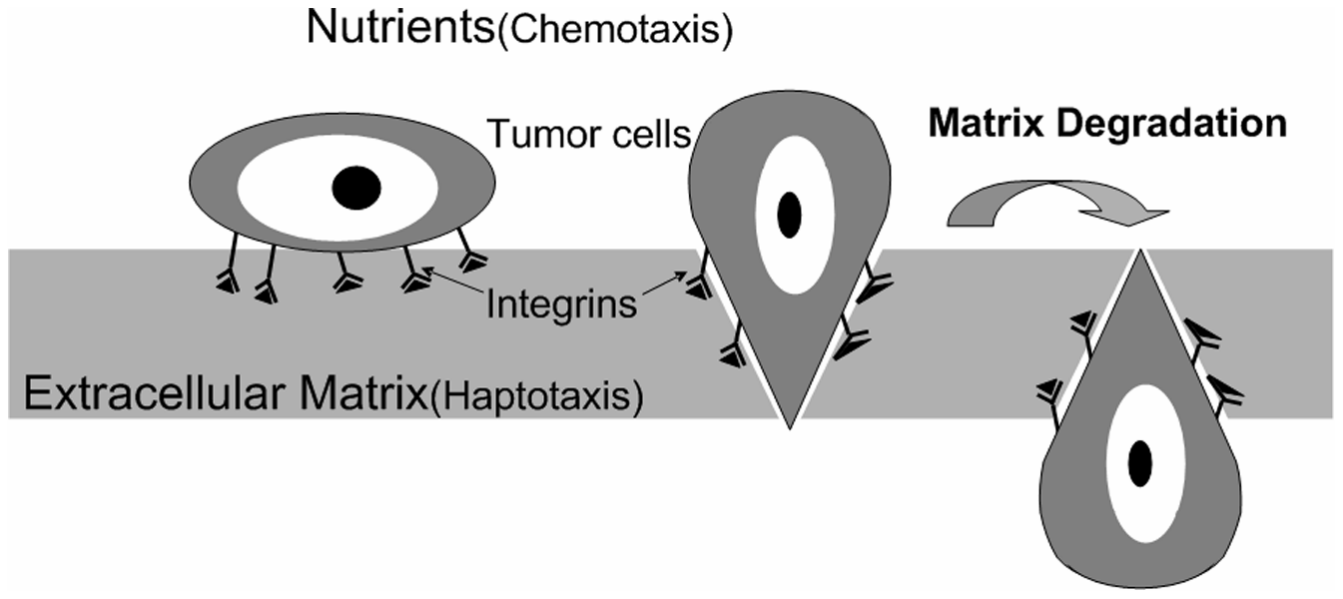
Schematic diagram of the host tissue surrounding a tumor in response to cell invasion. The tumor cells adhere to the surface of the ECM through integrins. Then, the tumor cells can invade into the ECM or surrounding tissue through the MMPs, which are ECM degradation enzymes secreted by the tumor cells [30], [31].

The schematic interactions between a tumor and the surrounding host tissue are shown in Fig. 1. Tumor cells interact with the ECM when a tumor begins to invade its host tissues. The tumor cells adhere to the surface of the ECM through integrins. The MMPs, which are ECM degradation enzymes secreted by the tumor cells, begin to degrade the ECM. The tumor cells can then complete their invasion of the ECM or surrounding tissue [38], [39]. The population density of tumor cells (*n*) quantifies the number of tumor cells per unit volume. The symbols *m* and *f* denote the MMP and the ECM concentrations, respectively. Provided that the outer cells shedding from a tumor are quite sparse, there is a sufficient supply of oxygen to a tumor. However, the outer cells are greatly dependent on the glucose concentration (*G*), which acts as an energy source in combination with the oxygen supply (the Warburg effect). The tumor cells tend to move up the glucose concentration gradient, which is referred to as chemotaxis [40]–[45].

The conservation equation for the tumor cell density *n* is:



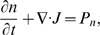
(1–1)where *J* is the flux of the cells and *P_n_* is a quantity relevant to both the proliferation and death of the cells. The flux *J* consists of four different parts:



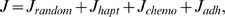
(1–2)where *J_random_, J_hapt_, J_chemo_, and J_adh_* are the fluxes contributed by random motion, haptotaxis, chemotaxis, and cell-cell adhesion, respectively. We assume that the ECM is a homogeneous medium so that tumor cells move in random motion; the *J_random_* is expressed in the form



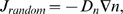
(1–3)where *D_n_* is the diffusive coefficient of the tumor cells [46]. Haptotaxis is the directed migratory response of cells to gradients of ECM, we assume that tumor cells are affected by the spatial gradient of *f*, the haptotaxis flux is given by



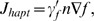
(1–4)where *γ'_f_* is the haptotaxis coefficient [46]. Tumor cells are strongly attracted to glucose G (the Warburg effect), and tend to move in the direction of the spatial gradient of G, hence, the chemotaxis flux is given by



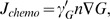
(1–5)where *γ'_G_* is the chemotaxis coefficient [19]. As discussed in cell-based models [19], [46]–[49] and observed in experiments, cells adhere to each other when they are close enough, but push apart when they are too compressed by neighboring cells. We expect that cells experiencing cell-cell adhesion are less likely to be able to move in regions of high cell density. We therefore assume that the adhesive flux is proportional to the density of the cells and the forces between them and inversely proportional to cell radius, *R*;



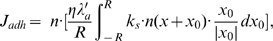
(1–6)where *λ'_a_* is the adhesion coefficient, *η* is the viscosity parameter, *k_s_* is a parameter that characterizes the adhesive force that the cells in the *R*- microenvironment exert at a point *x*, *x_0_* is the distance of cells away from the position *x*; the more details are described in the references by Armstrong et al. and Kim et al. [19], [46].

The term *P_n_* is related to the proliferation and death of cells and is formulated by the logistic growth function and the immune function [50], [51]:



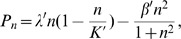
(1–7)where *λ'* is the growth rate of the tumor cell, *K'* is the carrying capacity of the environment [52], and *β'* is the immune coefficient [34].

The ECM is a deformable network of fibers degraded by the MMPs [53]. The degradation of the ECM is expressed by: 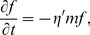
(2)where *η'* is the degradation coefficient of the ECM.

The MMPs are produced by the tumor cells. They diffuse with a constant diffusivity *D_m_* and decay at a linear rate, which is given by:



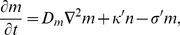
(3)where *D_m_* is the MMP diffusion coefficient, *κ'* is the MMP production rate, and *σ'* is the MMP decay coefficient.

Following Kim et al. [19], we assume that the concentration of glucose satisfies the reaction-diffusion equation:



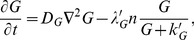
(4)where *D_G_* is the diffusion coefficient, *λ'_G_* is the rate of nutrient consumption by the tumor cells and *k'_G_* is a consumption parameter.

The PDEs (1-1), (2), (3), and (4) were solved numerically using the commercial software COMSOL Multiphysics on a square spatial domain *Ω* (a region of tissue) with appropriate initial and boundary conditions for each variable. We assumed that the tumor cells, ECM, and MMPs are confined within the domain of tissue under consideration and selected no-flux boundary conditions for the PDEs (1-1), (2), (3), and (4). We also assumed that the glucose concentration on the migrating boundary of the tumor is maintained invariably.

### Non-dimensionalization and parameterization

We performed a non-dimensionalization of the PDEs by rescaling the length with an appropriate scale *L* (e.g., the maximum invasion distance of cancer cells, which is approximately 1 cm for a tumor at the early stage of invasion), the time with *τ* (the average time of cell mitosis), the tumor cell density with *n_ref_*, the ECM density with *f_ref_*, the MMP concentration with *m_ref_*, and the glucose concentration with *G_ref_*. The symbols *L, τ, n_ref_, f_ref_, m_ref_*, and *G_ref_* are appropriate reference variables selected for reduction and they are specified in Table 1.

**Table 1:** Reference variables used in the tumor model.

Description		Dimensional value	Refs
*L*	Length	1 cm	[18], [62]; this work
*τ*	Time	8 h–24 h(16 h)	[18], [62]; this work
*n_ref_*	Reference tumor cell density	6.7×10^7^ cells⋅cm^−3^	[63]
*f_ref_*	Reference ECM concentration	10^−8^→10^−11^ M	[64]
*m_ref_*	Reference MMPs concentration	130 ng⋅ml^−1^	[65]
*G_ref_*	Reference Nutrient concentration	6.0×10^−4^ g⋅cm^−3^	[66]

We reduced the quantities in the PDEs (1-1, 2, 3, and 4) with the relevant reference variables:
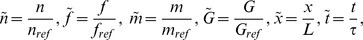
and dropped the tildes for notational convenience. We obtained the scaled system of equations:
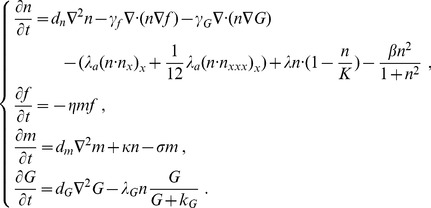
(5)with the boundary conditions:



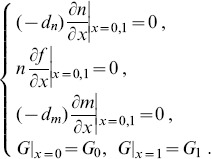
(6)Although the glucose concentration *G* remains invariably on the migrating boundary of the tumor, we set *G_1_>G_0_*, as more glucose is consumed at the core of a tumor (*x* = 0) than at the migrating boundary (*x = *1).

For the sake of simplification, we assumed that an initial tumor located at *x* = 0 spreads isotropically. The tumor's spread can therefore be reduced from two dimensions to one dimension, ranging from *x* = 0 with an initial density of *n(x,0)* to *x* = 1. We assumed that the tissue surrounding the tumor has been partially degraded by the tumor, that the initial concentration profile of the MMPs is proportional to the initial cell density and that the initial glucose concentration satisfies a Gaussian distribution. 
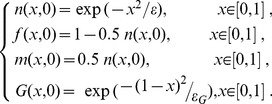
(7)where *ε* and *ε_G_* are the standard deviations of *m* and *G*, and *ε* and *ε_G_* are two positive parameters.

The new dimensionless parameters are: 
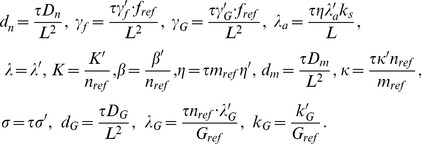
(8)


Table 2 lists the values used for the parameters in the subsequent simulations. Unless otherwise specified, the simulation space was discretized into 100 grids equally, and time steps were self-adjusted with a relative tolerance of 0.001.

**Table 2:** Parameters used in the tumor model.

Description		Dimensional value	Refs
Diffusion coefficient			
*D_n_*	Tumor cell	1×10^−11^ cm^2^/s	[19]
*D_m_*	MMP	(8±1.5)×10^−9^ m^2^/s(8×10^−9^)	[67]
*D_G_*	Nutrients(glucose)	1.3×10^−6^ cm^2^/s	[68]
Proliferation and immune parameters			
*λ'*	Tumor growth rate	1.0/τ	[50]
*K'*	The carrying capacity of the environment	10⋅n_0_	[50]
*β'*	Immune coefficient	2.2⋅n_0_/τ	[50]
Production, decay/consumption rates			
*η'*	Degradation of ECM	3.0×10^8^ cm^3^g^−1^s^−1^	[19]
*κ'*	the production rate of MMP	6.94×10^−8^ s^−1^	[19]
*σ'*	Natural decay of MMP	5.0×10^−6^ s^−1^	[19]
*λ'_G_*	Consumption rate of the nutrients by cells	2.56×10^−8^/n_0_ s^−1^	[19], [23], [69]
*k'_G_*	Nutrient consumption parameter	2.0×10^−3^ cm^3^/g	[19], [23], [69]
Haptotaxis, chemotaxis, and adhesion force parameter			
*γ'_f_*	Haptotaxis coefficient	2600 cm^2^ s^−1^ M^−1^	[63]
*γ'_G_*	Chemotactic sensitivity coefficient	2.76×10^−4^ cm^5^g^−1^s^−1^	[19]
*λ'_a_*	Strength of adhesion force between cells	(1×10^−5^→1×10^−3^)dyne(8×10^−3^)	[19]
*ε*	positive parameter	0.00025	[70]; this work
*ε_G_*	positive parameter	1.125	[70]; this work
*G_0_*	The reduced Nutrient concentration on the core of tumor	Exp<-(1-x)^2^/ε_G_>	[66], [71]; this work
*G_1_*	The reduced Nutrient concentration far from the tumor	1	[66], [71]; this work

### Diffusion type criterion

The diffusion type criterion was first proposed by Metzler et al. [54]. Based on the description of diffusion theory, the root-mean squared displacement (RMSD) of Brownian motion particles can be used to represent the random motion of particles over time. The RMSD usually relies on the evolution time (*t*), where RMSD ∼ *t^b^* and the power exponent *b* characterizes the nature of the diffusion process. Fickian diffusion corresponds to *b = 0.5*. If *0<b<0.5*, the diffusion is identified as subdiffusion, implying a slower diffusion process than Fickian diffusion. If *0.5<b<1.0*, the diffusion observed is superdiffusion and proceeds faster than Fickian diffusion. If b≥1.0, the diffusion is referred to as a ballistic diffusion, which is a peculiar characteristic of rapid, long-range motion.

### Methodology

We adopted the finite difference method to quantify the diffusion behavior of an isotropic tumor invasion in a one-dimensional space. The number density of the tumor cell (*n*) varies spatiotemporally with the evolution time (*t*) and spatial length (*x*). Two types of data, the peak location and the half-width of the *n* curve, were collected to quantify the diffusion process. The dependence of either the peak location or the half-width of the *n* curve on *t* was studied, depending on whether the shifting of the peak location or the broadening of the half-width dominated. Either the peak location or the half-width acts as a sort of diffusion front similar to the spatial RMSD. The power exponent *b* was calculated by fitting the relationship between the peak location of *n* and *t*, using a power function and the least squares method.

Both the *in vitro* cell cultures and clinical images were processed using the leading commercial image analytical software Image Pro Plus (IPP) for biology and medicine in order to extract outline of tumors precisely. The algorithms and source codes for measuring and calculating the mean radius of a tumor are presented in the supporting information (Text S1).

## Results

### Simulation results

To investigate the effects of the microenvironment around a tumor on the tumor's invasion, the terms representing ECM haptotaxis, glucose chemotaxis, tumor proliferation, immunization, and cell-cell adhesion were included singly and in combination in the set of PDEs (eq. 5). The general power exponents *b* calculated under each simulation condition (parameter) are summarized in Table 3 in advance for the convenience of subsequent comparison and discussion. The simulation results listed in Table 3 for different parameters will be detailed further in the subsections.

**Table 3:** The main parameters used in each simulation and the resulting calculated power exponent *b* values.

The main parameters	*b*	*Standard Error*	*Adj. R-Square*	Remarks
P1: *γ_f_ = γ_G_ = λ_a_ = λ = β = 0*	0.49618	0.00134	0.99960	Simple diffusion
P2: *γ_f_ = 0.01, γ_G_ = λ_a_ = λ = β = 0*	0.68564	0.00518	0.99860	Only haptotaxis
P3: *γ_f_ = 0, γ_G_ = 0.00828, λ_a_ = λ = β = 0*	0.52208	0.00334	0.99970	Only chemotaxis
P4: *γ_f_ = γ_G_ = 0, λ_a_ = 0.003, λ = β = 0*	0.40973	0.01406	0.98772	Only adhesion
P5: *γ_f_ = 0, γ_G_ = 0.00828, λ_a_ = 0.003, λ = β = 0*	0.46888	0.01722	0.98983	Chemotaxis + adhesion
P6: *γ_f_ = 0.01, γ_G_ = 0, λ_a_ = 0.003, λ = β = 0*	0.64126	0.00899	0.99869	Haptotaxis + adhesion
P7: *γ_f_ = 0.01, γ_G_ = 0.00828, λ_a_ = λ = β = 0*	0.72993	0.00581	0.99918	Haptotaxis + chemotaxis
P8: *γ_f_ = 0.01, γ_G_ = 0.00828, λ_a_ = 0.003, λ = β = 0*	0.66582	0.01038	0.99839	Haptotaxis + chemotaxis + adhesion
P9: *γ_f_ = 0.01, γ_G_ = 0.00828, λ_a_ = 0, λ = 1.0, β = 2.2*	0.87903	0.04864	0.98902	All of the effects except adhesion
P10: *γ_f_ = 0.01, γ_G_ = 0.00828, λ_a_ = 0.003, λ = 1.0, β = 2.2*	0.85377	0.05778	0.98327	All of the effects
P11: *γ_f_ = 0.01, γ_G_ = 0.00828, λ_a_ = 0.003, λ = 0.5, β = 2.2*	0.70455	0.026804	0.99443	All of the effects(a weaker proliferation rate)
P12: *γ_f_ = 0.01, γ_G_ = 0.00828, λ_a_ = 0.003, λ = 1.0, β = 4.4*	0.77814	0.041857	0.98915	All of the effects(a stronger immunization)
P13: *γ_f_ = 0.08, γ_G_ = 0.06, λ_a_ = 0, λ = 2.8, β = 1.1*	1.36420	0.08157	0.99392	All of the effects except adhesion (very malignant)
P14: *γ_f_ = 0.08, γ_G_ = 0.06, λ_a_ = 0.00003, λ = 2.8, β = 1.1*	1.35020	0.08457	0.99373	All of the effects (very malignant, weak adhesion)

#### Tumor invasive diffusion without the effects of ECM haptotaxis, glucose chemotaxis, tumor proliferation, immunization, and cell-cell adhesion

**Figure 2:**
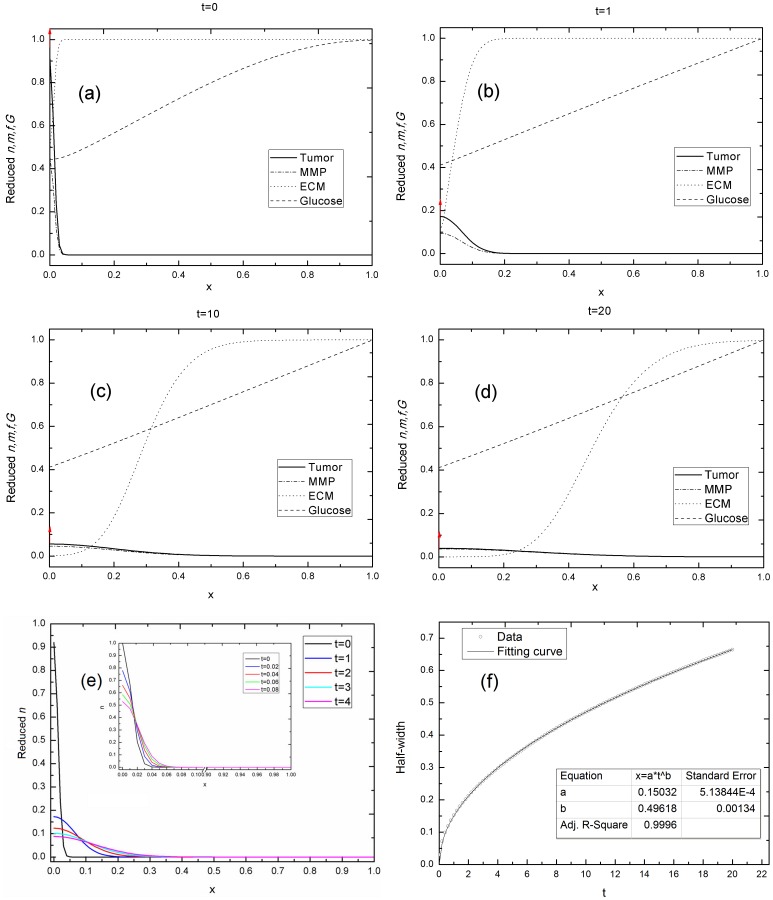
Tumor invasion is unaffected by the microenvironment. (a–d) The time evolution of the *n*, MMP, ECM, and glucose in a one-dimensional spatial length *x* at time (a) *t = 0*, (b) *t = 1*, (c) *t = 10, and* (d) *t = 20*. (e) The cell density *n(x,t)* when *t* ranges from *0* to *20*. The inset shows *t* ranging from *0* to *0.08*. (f) The curve fitting the half-width of cell density versus evolution time data, *x_h_-t, b = 0.49618±0.00134*. Simulation parameters: *γ_f_ = γ_G_ = λ = β = λ_a_ = 0*. The rests of the parameters were fixed. The ECM is degraded by the MMP; the simulated exponent *b* in this situation is almost *0.5*, the typical Fickian diffusion.

P1 in Table 3 represents tumor invasion unaffected by the microenvironment (surrounding host tissue). The resulting spatial variations in the *n*, MMP, ECM, and glucose at the times *t = 0*, *1*, *10*, and *20* are plotted in Figs. 2(a)–(d), respectively. According to Fig. 2(e), a broadening of the half-width of the *n* curve, not a shifting of the peak locations of the *n* curve, is observed after different evolution periods. Therefore the half-width, instead of the peak locations of the *n* curve, was used to quantify the tumor's diffusion. The half-width increases with the evolution time, as indicated in Fig. 2(f). The power exponent *b* was calculated by fitting a curve to the half-width versus time data, using a power function *∼ t^b^* and the least squares method. The power exponent *b = 0.49618±0.00134* was obtained, which is very close to the Fickian diffusion index *b = 0.5*. The tumor invasion in this situation is therefore characteristic of normal diffusion satisfying the Einstein relationship.

The trend of the ECM curve obviously reflects MMP degradation, because the ECM concentration declines as the MMP concentration increases at the same location, but at a later time. The macromolecules within the ECM are readily degraded by different proteolytic enzymes, which are the MMPs [55]. Therefore the ECM can be hydrolyzed distinctly when the MMPs permeate it.

#### Tumor invasive diffusion with haptotaxis and heterogeneity of the ECM

**Figure 3:**
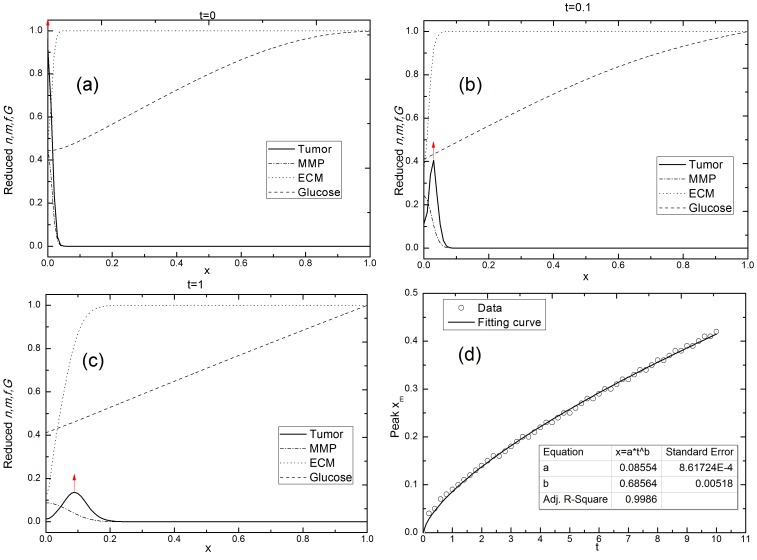
Tumor invasive diffusion with haptotaxis of the ECM. (a–c) The time evolution of the *n*, MMP, ECM, and glucose in a one-dimensional spatial length *x* at (a) *t = 0*, (b) *t = 0.1*, and (c) *t = 1*. (d) A power function curve is fitted to the time and peak position of *n*, *x_m_-t*, *b = 0.68564±0.00518*. Simulation parameters: The haptotaxis coefficient *γ_f_ = 0.01* and *γ_G_ = λ = β = λ_a_ = 0*. The rests of the parameters were fixed. The peak location of the *n* curve shifts considerably with the evolution time when haptotaxis of the ECM is included and the exponent *b* is larger than *0.5*, signifying occurrence of superdiffusion.

Haptotaxis and the heterogeneity of the ECM play an important role in the diffusion process of a tumor invasion. Figs. 3(a–d) show the variations in the *n*, MMP, and ECM at different times under the condition P2 in Table 3. The tumor invasion here is obviously different from the situation with no ECM. The peak location of the *n* curve shifts considerably with the evolution time when haptotaxis and the heterogeneous distribution of the ECM are included. The shifting of the peak location reflects the proliferative invasion of a tumor into the surrounding tissue, as the peak of *n*, which behaves as a sort of diffusion front, migrates into the interior of the ECM. The ECM concentration varies drastically near its border with the tumor when haptotaxis is included, indicating that coupling between the ECM and the tumor cell can expedite ECM degradation by the MMPs. Accompanying this degradation, the tumor cells invade using a combination of diffusion and haptotaxis. The peak of the tumor cell density *n* at *t = 0.1* and *1* indicates that a cluster of tumor cells forms on the diffusion front of a spreading tumor due to haptotaxis migration, which is consistent with Anderson et al. [18], [19], [39]. This phenomenon can also be verified by *in vitro* cell cultures and clinical observations, which will be described later in this article.

**Figure 4:**
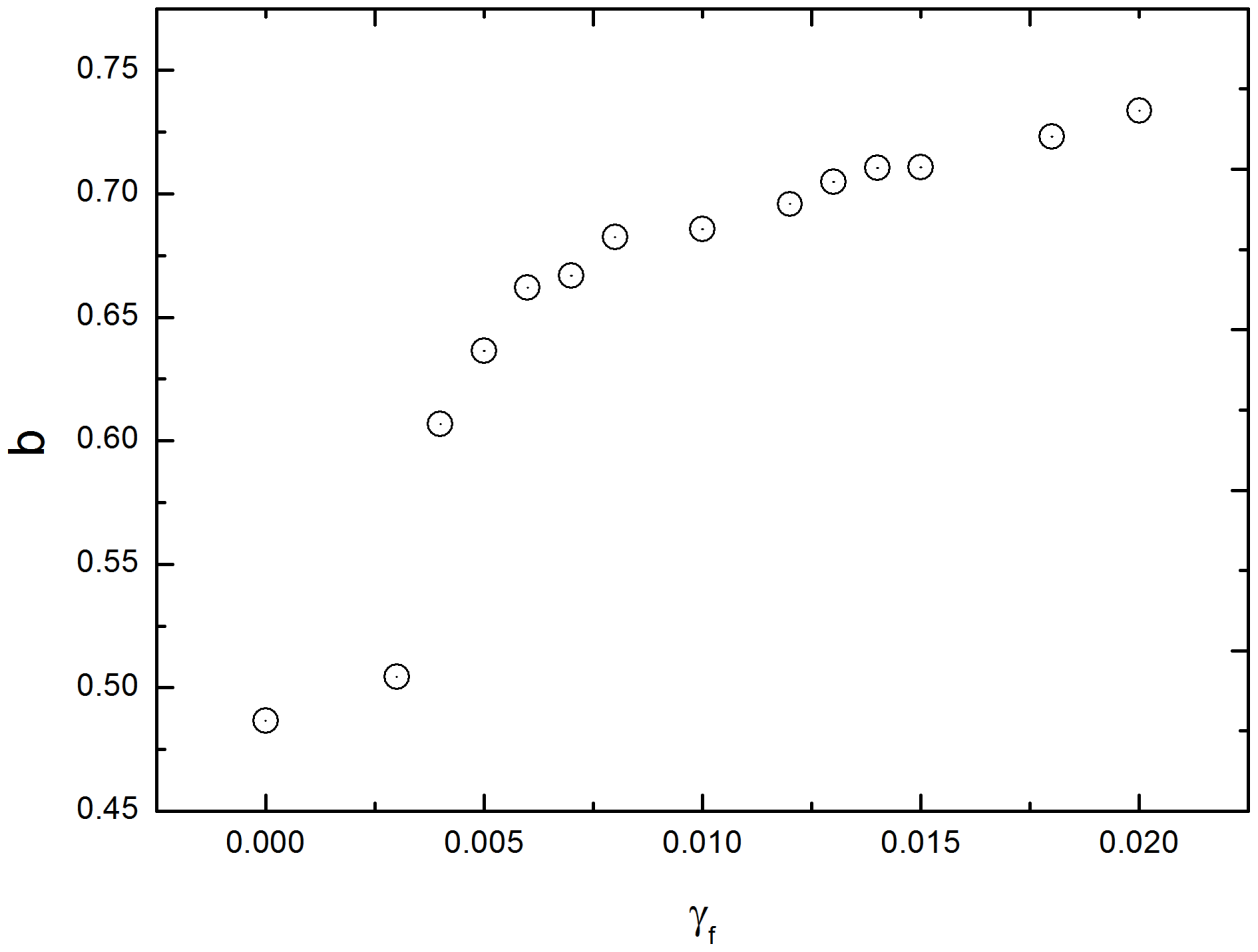
The simulated exponent *b* with a variety of haptotaxis coefficients *γ_f_*. The *b* increases with the *γ_f_*, indicating that a tumor's invading is enhanced by haptotaxis of the ECM. Simulation parameters: *γ_G_ = λ = β = λ_a_ = 0*. The rests of the parameters were fixed.

A further simulation demonstrates that the power exponent *b* increases with the haptotaxis coefficient *γ_f_* (Fig. 4), which agrees with Kim et al. [19]. The exponent *b* is larger than 0.5 in the presence of the ECM surrounding a tumor. The diffusive invasion of a tumor that experiences haptotaxis toward its host ECM is therefore superdiffusion, implying a more rapid diffusion.

According to the previous studies [56], [57], the homogeneity of the ECM can affect cell migration. We investigated the effect of the heterogeneity of the ECM on a tumor's invasive diffusion using a simple heterogeneous distribution of the ECM, changing the initial conditions of the ECM distribution as follows: 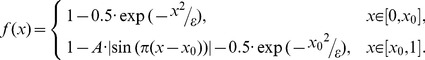
(9)where*x_0_ = 0.05*, because the ECM is of a uniform distribution when *x>0.05* (Fig. 3(a)) and *A* is a dimensionless parameter. Increasing A's value gives a more heterogeneous ECM distribution. The initial distribution is adjustable, to obtain a non-uniform distribution of the ECM when *x>0.05*.

**Figure 5:**
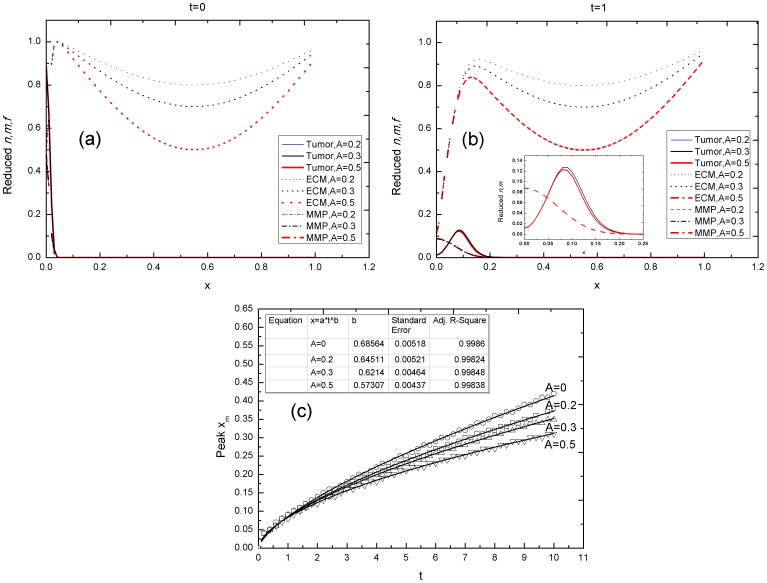
Tumor invasive diffusion with heterogeneity of the ECM. (a–b) The time evolution of the *n*, MMP, and ECM in a one-dimensional spatial length *x* when the ECM is heterogeneous, at times (a) *t = 0* and (b) *t = 1*. The inset shows the enlarging curves of the *n* and MMP at a different time. (c) The curve fitting the time and peak position of *n* data, *x_m_-t*, at different values of A. The exponent *b* decreases with the heterogeneity of the ECM. Simulating parameters: P2 in Table 3, where *γ_f_ = 0.01* and *γ_G_ = λ = β = λ_a_ = 0*. The rests of the parameters were fixed.

Figs. 5(a) and (b) show the influence of the heterogeneous distribution of the ECM on a tumor invasion. In Fig. 5(c), the peak position *x* of the cell density *n* and time *t* data are fitted by *x∼t^b^*. According to Fig. 5(c), the exponent *b* decreases with the heterogeneity of the ECM, but still remains larger than 0.5. This result indicates that either a heterogeneous ECM distribution or a lower ECM concentration weakens haptotaxis toward the ECM, but that diffusion is still faster than when haptotaxis is excluded. Judging by the value of the exponent *b*, the tumor invasion in this case is also superdiffusive.

#### The effect of glucose chemotaxis on the diffusion process of tumor invasion

**Figure 6:**
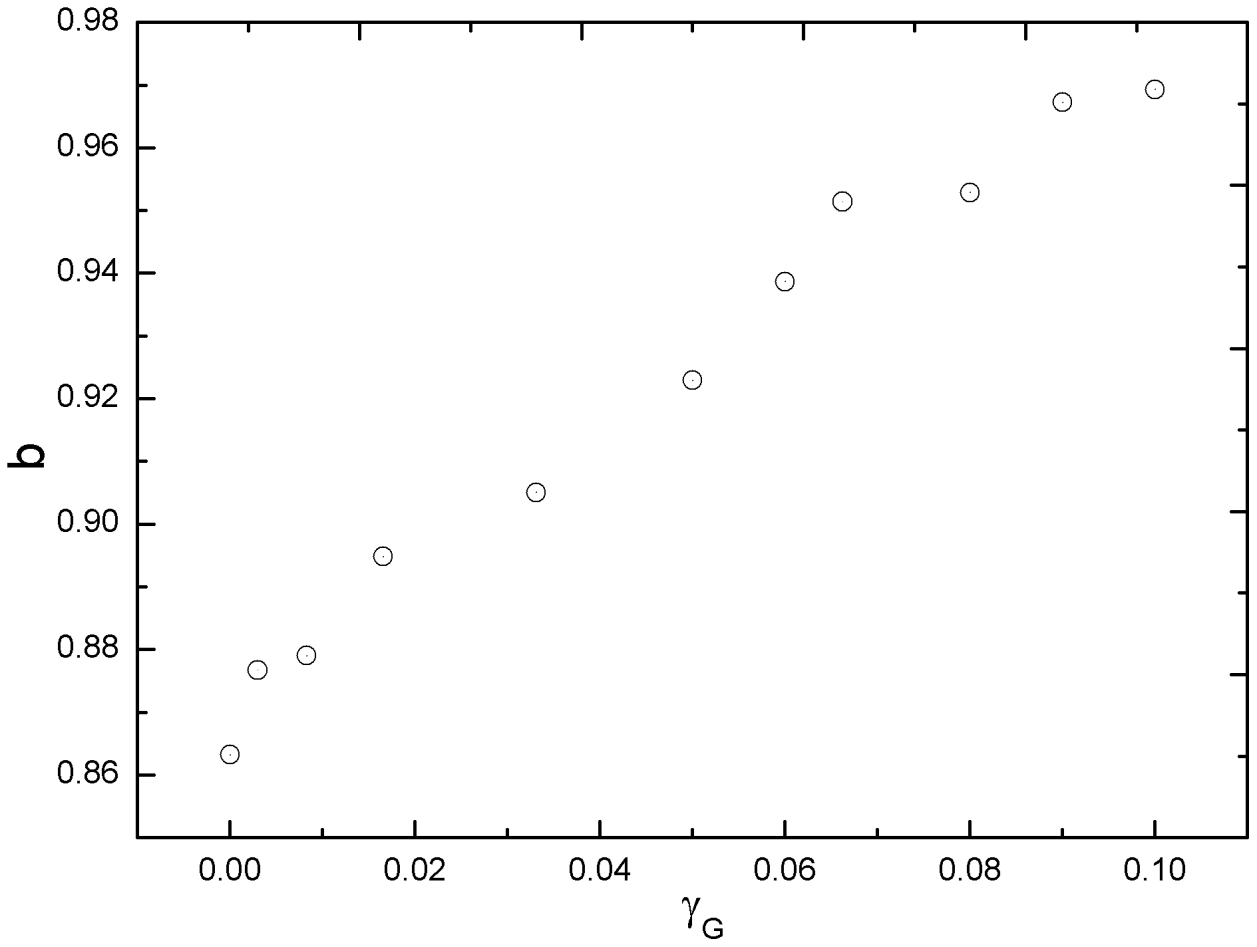
The simulated exponent *b* with a variety of chemotaxis coefficients *γ_G_*. The *b* increases with the ***γ_G_***, implying that a tumor's invading is promoted by glucose chemotaxis. Simulation parameters: *γ_f_* = 0.01, *λ = 1.0*, *β = 2.2*, and *λ_a_ = 0*. The rests of the parameters were fixed.

The same method was used to evaluate the effect of glucose chemotaxis on the diffusion process of a tumor invasion. The ECM haptotaxis coefficient, adhesion coefficient, tumor growth rate and immune coefficient were kept constant. Table 3 lists the parameters (P3 glucose only, P9) and results. The simulation results are given in the supporting information (File S1). The values for the exponent *b* based on P9 versus various chemotaxis coefficients are given in Fig. 6. Including the effects of chemotaxis toward the glucose source surrounding a tumor (*γ_G_≠0*) raises the exponent *b* and the superdiffusive tumor invasion is enhanced further by chemotaxis toward glucose. According to the criterion of diffusion, chemotaxis toward glucose promotes a tumor invasion, resulting in superdiffusion.

#### The effect of cell-cell adhesion on the diffusion of a tumor invasion

**Figure 7:**
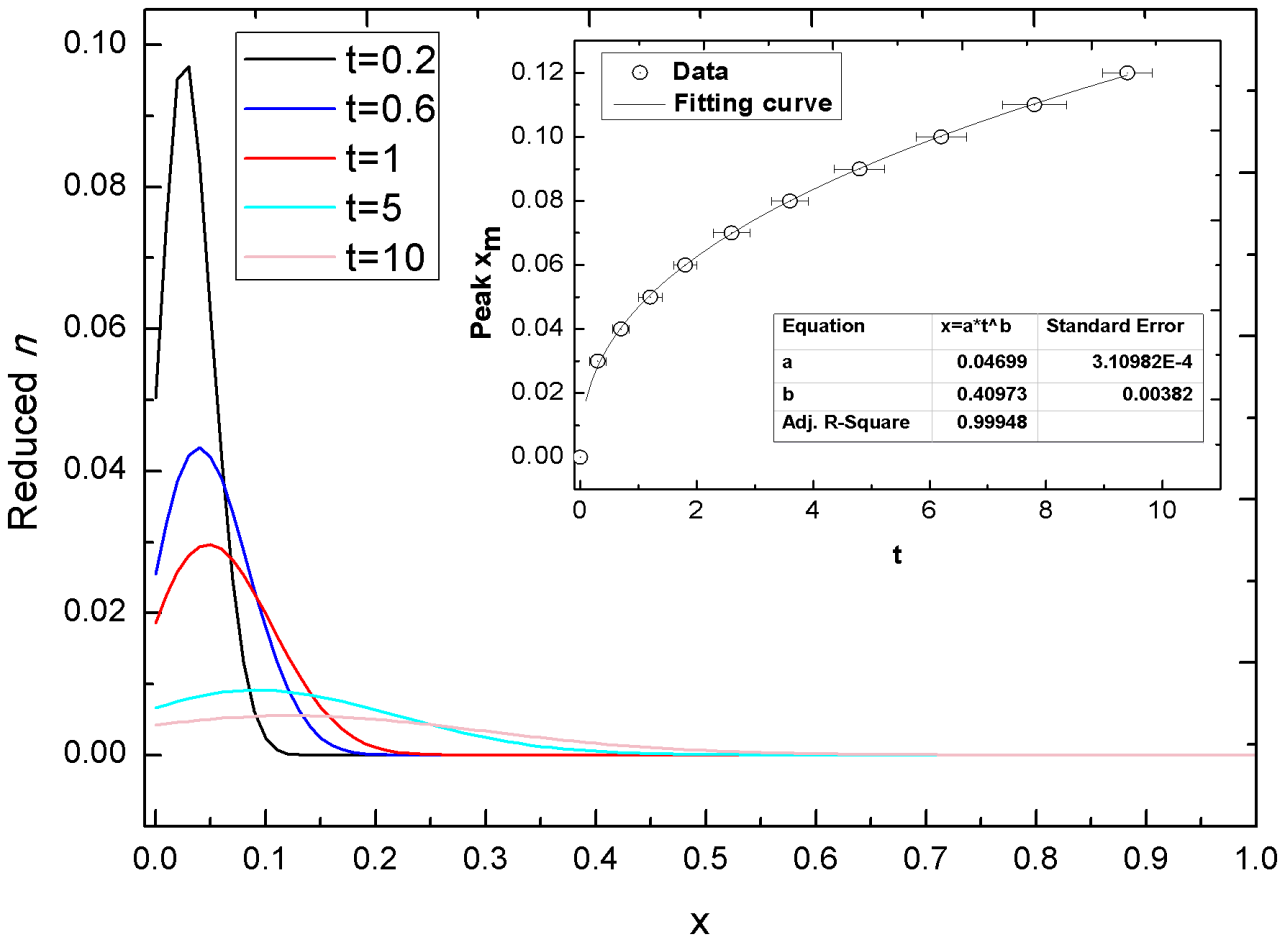
The time evolution of the *n* at different times. The inset is the curve fit to the time and peak position of the cell density data, *x_m_-t*, *b = 0.40973±0.00382*, where *λ_a_ = 0.003*. Parameters: *γ_f_ = γ_G_ = λ = β = 0*, the rests of the parameters were fixed. The exponent *b* is smaller than *0.5*, signifying occurrence of subdiffusion.

The influence of cell-cell adhesion on the diffusion of a tumor invasion was also explored. The results are shown in Table 3 (P4, P5, P6, P8, P10, and P14). When the effect of only cell-cell adhesion is included in the microenvironment of a growing tumor (P4, Fig 7), the exponent *b* is 0.40973, smaller than 0.5, indicating that the diffusion behavior of a tumor invasion in pure cell-cell adhesion state is of subdiffusion. Diminishing the diffusive invasion of a tumor by cell-cell adhesion could also be identified in other situations involving cell-cell adhesion (P5, P6, P8, P10, and P14), as compared to their counterparts without cell-cell adhesion.

### The experimental results

**Figure 8:**
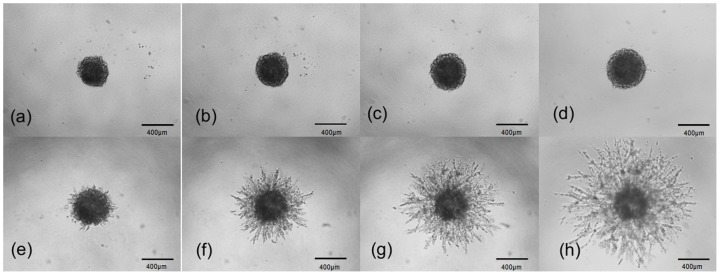
The images are taken from *in vitro* culturing of cells from the breast line MDA-MB-231. Upper row: MDA-MB-231 cells without a matrix on the (a) 1st day, (b) 2nd day, (c) 3rd day, and (d) 4th day. Bottom row: MDA-MB-231 cells with a matrix on the (e) 1st day, (f) 2nd day, (g) 3rd day, and (h) 4th day. All cells were maintained in DMEM 10 µg/ml gentamycin at 37°C in 5% CO2, the matrix was derived from murine EHS sarcoma cells and collagen. (a–h) reprinted figures with permission from Trevigen Inc (Cultrex Catalog #: 3500-096-K).

**Figure 9:**
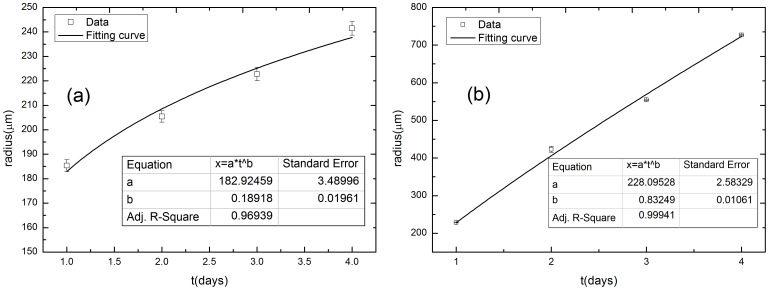
The average radius of MDA-MB-231 cancer cell cultures versus culture time. (a) MDA-MB-231 cells without a matrix and (b) MDA-MB-231 cells with a matrix. The exponent *b* is larger than 0.5 for the cells with a matrix and smaller than 0.5 for the cells without a matrix.

A cell culture invasion assay provided a physiological approach for assessing tumor invasion and offered a visual component that could be quantified through image analysis. Preliminary results from the *in vitro* culturing of cancer cells and clinical imaging of cancer patients were used to verify the simulation results. We measured the size of a tumor at different evolution times and determined the fractal dimension of the periphery between a growing tumor and the surrounding tissue from both the *in vitro* cultures and clinical images. Figs. 8 show representative images of growing cultured cells (MDA-MB-231) without a matrix (a∼d) and with a matrix (e∼h). The cultured cells with a matrix appear to invade the surrounding matrix with a more open, dendritic diffusion front, whereas the cells without a matrix simply spread with a closed smooth diffusion front. The average radius of the two sets of cells over time is plotted in Fig. 9 and fitted with the power function *t^b^*. For these b values the statistical errors were estimated from three independent measurements. Prevalence estimates are reported with corresponding 95% logit confidence intervals (CI). The exponent *b* is 0.18918±0.01961 for the cells without a matrix and 0.83249±0.01061 for the cells with a matrix. The exponent *b* is larger than 0.5 in the culture of MDA-MB-231 cells with a matrix and smaller than 0.5 in the culture of MDA-MB-231 cells without a matrix. A tumor can therefore spread either rapidly or slowly, depending on whether the matrix surrounding the tumor is involved. The same method was used to analyze other cell lines and the results are listed in Table 4. The materials information of cell cultures is shown in the supporting information (File S2). The data and curve fitting are shown in the supporting information (File S3).

**Table 4:** The measured value of the exponent *b* for different *in vitro* tumor cells with and without the ECM.

Cell line	*b*	*Standard error*	*Adj. R-square*	Remarks
A549	0.12621	0.00974	0.93309	Without matrix
SiHa HCC	0.39328	0.05153	0.94231	Without matrix
MDA-MB-231	0.18918	0.01961	0.96939	Without matrix
MDA-MB-231	0.83249	0.01061	0.99941	With matrix
U87 MG(ULA)	0.6439	0.03858	0.9895	With matrix
U87 MG(agar)	0.8988	0.06269	0.98948	With matrix

Judging by the diffusion type criterion and the results in Table 4, the diffusion of the cell cultures with a matrix can be identified as superdiffusion and without a matrix as subdiffusion. The *in vitro* results in Table 4 conform to the simulation results presented above that show that ECM haptotaxis accelerates a tumor invasion.

**Figure 10:**
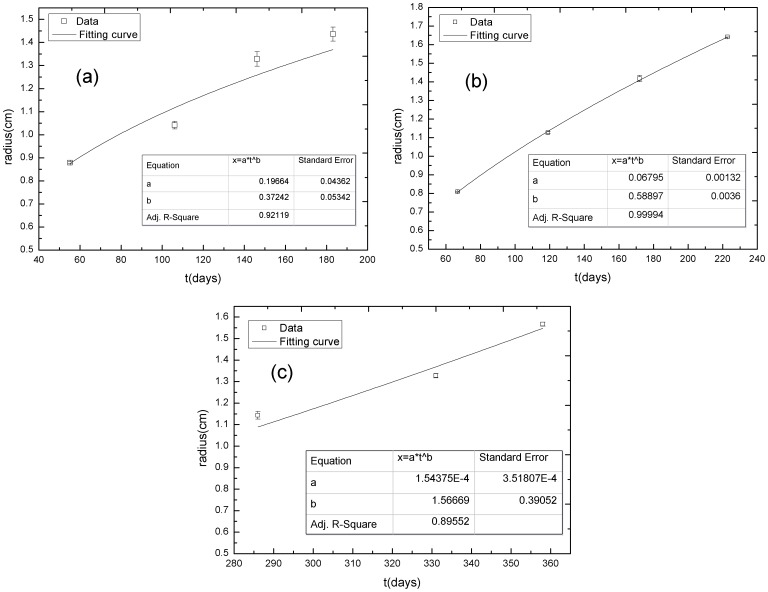
The relationship between tumor size (*r*) and time (*t*) in clinical tumors. (a) the adrenal tumor, (b) liver tumor A, and (c) liver tumor B. The exponent *b* of the adrenal tumor is smaller than 0.5; the *b* is between 0.5 and 1.0 for liver tumor A and larger than 1.0 for liver tumor B.

Clinical image data were also included to account for the spreading of a tumor in a clinical diagnosis. The Sun Yat-sen University Cancer Center provided data from three cancer patients who had been clinically diagnosed with a metastatic adrenal tumor (patient 1 with an expansive growing tumor) or a metastatic liver tumor (patient 2 with an infiltrative growing tumor, patient 3 with three infiltrative growing tumors). The clinical images data and curve fitting are shown in the supporting information (File S4). For the sake of convenience, the liver tumor lesions in the two patients were numbered tumors A, B, C, and D (liver tumor A is the liver metastasis from choroidal melanoma with low-grade malignancy; liver tumor B, C, and D are the liver metastases from colorectal cancer with high-grade malignancy). The adrenal tumor demonstrated a typical expansive growth, whereas the liver tumors displayed invasive spreading. The tumor sizes were measured and the size-time relationships are displayed in Fig. 10. The power exponent b was calculated using the same route as before. The exponent b and statistical data are listed in Table 5.

**Table 5:** The calculated values of the exponent *b* for three tumor patients.

Clinical data	*b*	*Standard Error*	*Adj. R-Square*	Remarks
**Patient 1: Adrenal tumor**	0.37242	0.05342	0.92119	Expansive growth
**Patient 2: Liver tumor A**	0.58897	0.00360	0.99994	Infiltrative growth with low-grade malignancy
**Patient 3: Liver tumor B**	1.56669	0.39052	0.89552	Infiltrative growth with high-grade malignancy
**Patient 3: Liver tumor C**	2.90653	0.23663	0.98801	Infiltrative growth with high-grade malignancy
**Patient 3: Liver tumor D**	2.24941	0.71183	0.83540	Infiltrative growth with high-grade malignancy

According to the power exponent *b* listed in Table 5 and the migrating patterns of the tumors seen in the clinical images, the adrenal tumor demonstrates an expansive but slow growth characteristic of subdiffusion, whereas the invasive liver tumors spread faster, characteristic of superdiffusion or even ballistic diffusion. It is not difficult to interpret the phenomenon that an invasive tumor can spread faster than an expansive tumor, as a spreading invasive tumor can break through the enclosure of basement membrane surrounding the tumor with sufficient nutrient exchange between the tumor and its surrounding host tissue. The peripheral border of an invasive tumor will be more open and rougher than an expansive tumor, which facilitates tumor invasion into the surrounding host tissue, especially when subjected to haptotaxis or chemotaxis stimuli from the ECM or nutrients.

**Figure 11:**
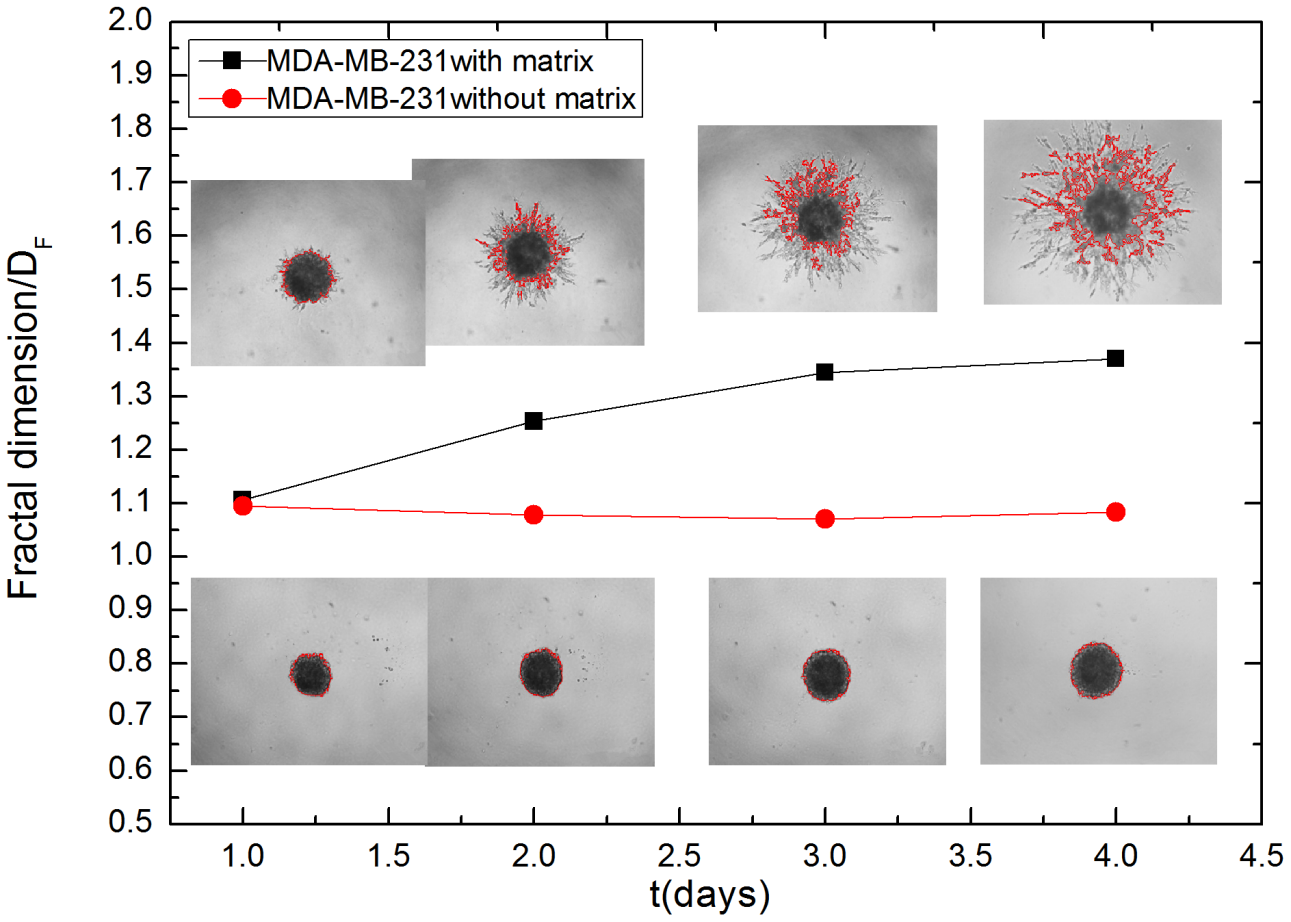
The border fractal dimensions of the *in vitro* cultured MDA-MB-231 cells. The border fractal dimension of the *in vitro* cells cultured with a matrix is larger than that of the cells cultured without a matrix.

Fractal analysis is a convenient, accurate method available for evaluating the roughness of a tumor border [58]–[61]. The border fractal dimensions of both the *in vitro* cultured cells and the clinical tumors were measured. The border fractal dimensions of the *in vitro* cultured cells are shown in Fig. 11. The border fractal dimension of the *in vitro* cells cultured without the ECM remains fixed at 1.1, whereas the cells cultured with the ECM rises from 1.1 to 1.4, accompanying the dendritic growth on the border between the cultured cells and the ECM. The dendritic growth pattern of a proliferating tumor also serves as an indicator that the tumor is penetratively invading the surrounding host tissue.

**Figure 12:**
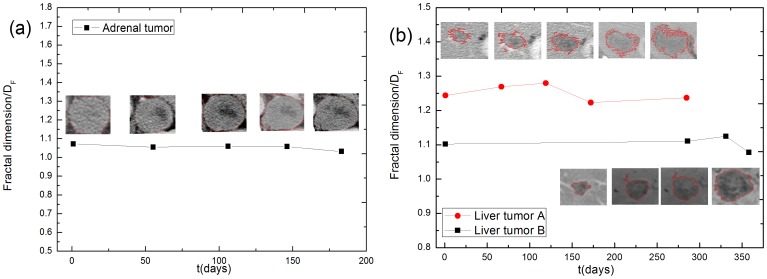
The border fractal dimensions of the clinical tumors. (a) The border fractal dimensions of the adrenal tumor. (b) The border fractal dimensions of the liver tumors A and B, analyzed using clinical medical images. The border fractal dimension remains at a low level for the adrenal tumor and maintains a high level for the infiltrative liver tumor *A*. The liver tumor *B* is clinically diagnosed as a metastatic tumor that has separated from its primary surrounding host tissue.

The border fractal dimensions of the clinical tumors, measured using medical images, of selected tumor patients are displayed in Fig. 12. The growth of the expansive adrenal tumor is confined inside its clear-cut basement membrane and its border fractal dimension remains at a low level (1.05∼1.1) during growth. In contrast, the border fractal dimension of the infiltrative liver tumor *A* maintains a high level (approximately 1.25) during invasion and the invasive border is blurred and rougher than in the expansive tumor. The liver tumor *B* is clinically diagnosed as a metastatic tumor that has separated from its primary surrounding host tissue. Its border fractal dimension is smaller than that of its primary counterpart, as its border remains close and smooth.

## Discussion

We combined mathematical modeling and experimental validation approach to uncover the diffusion processes that occur in tumor invasion under different growth microenvironments. The effects of ECM haptotaxis, glucose chemotaxis, cell-cell adhesion, tumor proliferation, and immunization on the diffusion of a tumor invasion were investigated. We extended the existing models of tumor cell migration [18], [19] by including haptotaxis, chemotaxis, logistic growth, and immunity [50], [51] to address the spatiotemporal growth of a tumor under more realistic conditions.

Tumor proliferation was found to have a positive role and immunization a negative role in tumor invasion. An increase in the tumor growth rate accelerates the diffusion process of a tumor invasion (P10, P11 in Table 3), whereas enhancing the intensity of the immune response counteracts a tumor invasion (P10, P12 in Table 3).

The ECM surrounding a tumor plays a crucial role in the diffusion of a tumor invasion, as shown in the simulations, *in vitro* tumor cell cultures, and clinical medical image analysis. Outer tumor cells that are subject to ECM haptotaxis can easily shed the tumor core and invade ECM-rich locations, displaying a peak of tumor cell density inside the ECM (Figs. 3(b) and (c)). The invasion of a tumor is accelerated by the tractive effect of ECM haptotaxis and the tumor cells infiltrate into the ECM superdiffusively. Both the simulations and *in vitro* cell cultures suggest that a larger ECM haptotaxis effect facilitates diffusion, leading to a faster tumor invasion over a wide range. Combining these results with a border fractal dimension analysis, we gained insights into the relationship between ECM haptotaxis and the border fractal dimension of a growing tumor. An increase in the border fractal dimension shows that the openness and roughness of the tumor border are considerably promoted by the ECM, revealing an active interflow of mass and energy between the tumor and its neighboring host tissue. The active interactions between a tumor and its surrounding host tissue, namely haptotaxis and chemotaxis in this study, intensify the diffusion of a tumor invasion.

Heterogeneity of the distribution of the ECM influences a tumor invasion considerably, by weakening the implantation of tumor cells within the ECM and limiting the auxo-action of the ECM on tumor invasion. The implantation of tumor cells, however, takes place only as long as the ECM surrounding the tumor is involved (Fig. 5). Moreover, tumor diffusion is strongly dependent upon the lesion location. A malignant tumor that is adjacent to rich peripheral vessels, such as liver tumors B, C, and D, diffuses rapidly and the power exponent *b* is even larger than 1.0 (Fig. 10(c), Table 5), representing ballistic diffusion (P14 in Table 3, Table 5) with distinctive rapid, long-range jumps by which diffusive particles separate themselves from the diffusion source and move away quickly. In clinical diagnosis, the migration of a metastatic tumor behaves in a similar way. We therefore identified a tumor with a power exponent *b* larger than one, i.e. a ballistic diffusive tumor, with a metastatic tumor. The power exponent *b* serves as an indicator of tumor invasion and metastatization.

When the glucose source surrounding a tumor is considered, the extreme thermodynamic costs of the oxidative Warburg cycle consumed by tumor cells drive the cells to move up the resulting glucose concentration gradient [44], using chemotaxis. The mobility of tumor cells is enhanced by chemotaxis. Therefore, the tumor invasion speeds up to a more apparent superdiffusion when the glucose consumption of a tumor is taken into account (P3 and P9 in Table 3, Fig. 6).

When either cell-cell adhesion or cell-cell adhesion plus dilute glucose are taken into account, the tumor invasion is a subdiffusive process (P4 and P5 in Table 3). Our simulation confirmed that cell-cell adhesion weakens tumor diffusion, resulting in a smaller exponent *b* (P4 and P5 in Table 3), but still contributes to the formation of a tumor cell density peak, which is a cluster of tumor cells forming on the diffusion front of a spreading tumor (Fig. 7). Cell-cell adhesion maintains the form of the cell cluster and prevents dispersion. The effect of cell-cell adhesion was complicated here, because both the form of cell cluster and the movement of cell cluster itself in space were taken into account simultaneously. Liu et al. [44] regarded cell invasion as an alternation process between adhesion and the elimination of adhesion. According to Liu et al. [44], cell-cell adhesion occurs at the leading front cells. The posterior cells adhesively follow the leading cells. New cells continuously take over the leading positions and the invasion front is continuously refreshed. Tumor cells can obtain a tractive effect from the process of alternating between adhesion and elimination of adhesion, which allows them to finally move to the front. Our simulation indicates that cell-cell adhesion not only contributes to the form of the cell cluster at the invasion front of the tumor, but also hinders the mobility of the invasion front in a subdiffusive way.

In conclusion, a tumor invasion is a complicated diffusion process and is strongly dependent on the surrounding microenvironment where the tumor is located. Our study used simulation, *in vitro* cell culture, and clinical medical imaging to reveal that a tumor invasion, depending on the status of the surrounding host matrix, can follow a broad spectrum of anomalous diffusion, ranging from subdiffusion to superdiffusion and ballistic diffusion, when haptotaxis and chemotaxis are involved. We now summarize our main findings. (1) A parameter referred to as the power exponent *b* was devised to characterize the proliferation and diffusion of an invasive tumor. (2) Both haptotaxis, initiated by the ECM and chemotaxis toward the glucose nutrient supply promote tumor invasion and lead to superdiffusive and even ballistic diffusive tumor invasion. We identified superdiffusion (0.5<*b*<1.0) and ballistic diffusion (*b≥1.0*) with the invasion and metastatization, respectively, of a malignant infiltrative tumor. (3) Cell-cell adhesion contributes to the form of the cell cluster at the invasion front of a tumor and hinders the mobility of the invasion front in a subdiffusive way. (4) The border fractal dimension of a tumor is an indicator for the openness and roughness of the tumor border. It is considerably promoted by the ECM, implying the occurrence of a more active interflow of mass and energy between a tumor and the neighboring host tissue.

It should be emphasized that the method that we introduced in this work to classify the diffusion of a tumor invasion needs further more verifications by tumor biology and clinical medicine before the power exponent *b* is acceptable as a practical indicator for characterizing tumor invasion and metastatization.

## Supporting Information

Text S1 **Matlab file to analysis the boundary of a cancer in an image.** The algorithms and source codes are used for measuring and calculating the mean radius of a tumor.
(TXT)

File S1 **The simulation results of P1–P14 in** **Table 3** **.**
(DOC)

File S2 **The materials and Methods information of cell cultures.**
(DOC)

File S3 **The data and curve fitting of A549 cell line, SiHa HCC cell line, U87 MG cell line (ULA), and U87 MG cell line (agar).** The A549 cell line was cultured without matrix, and the original images can be found in Essen BioScience Inc, Catalog Number: 4491 [1]; the SiHa HCC cell line was also cultured without matrix. Please refer to Kim's work [2] for the original data; both of the U87 MG cell lines were cultured with matrix, and the original data can be found in Vinci's work [3].
(DOC)

File S4 **The original graphs and curve fitting of Clinical images.** The Sun Yat-sen University Cancer Center provided data from three cancer patients who had been clinically diagnosed with a metastatic adrenal tumor (patient 1 with an expansive growing tumor) or a metastatic liver tumor (patient 2 with an infiltrative growing tumor, patient 3 with three infiltrative growing tumors). The liver tumor lesions in the two patients were numbered tumors A, B, C, and D (liver tumor A is the liver metastasis from choroidal melanoma with low-grade malignancy; liver tumor B, C, and D are the liver metastases from colorectal cancer with high-grade malignancy). This study was approved by the ethics committee of Sun Yat-sen University Cancer Center and protected the patients' private information.
(DOC)
