# Supplementary material for: The Anomalous Diffusion of a Tumor Invading with Different Surrounding Tissues
Source: PLoS One. 2014 Oct 13;9(10):e109784. doi: 10.1371/journal.pone.0109784 (PMC4195689; doi:10.1371/journal.pone.0109784)

**S1 The simulation results of P1 – P14 in Table 3**

P1: *γ_f_=γ_G_=λ_a_=λ=β=0*


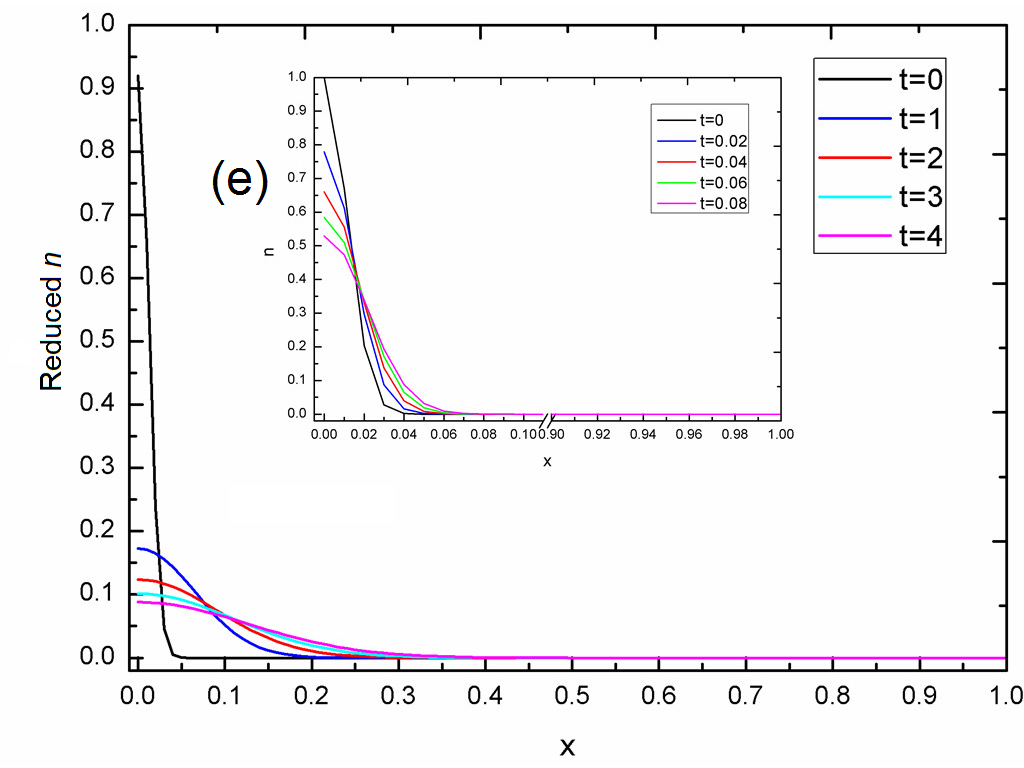

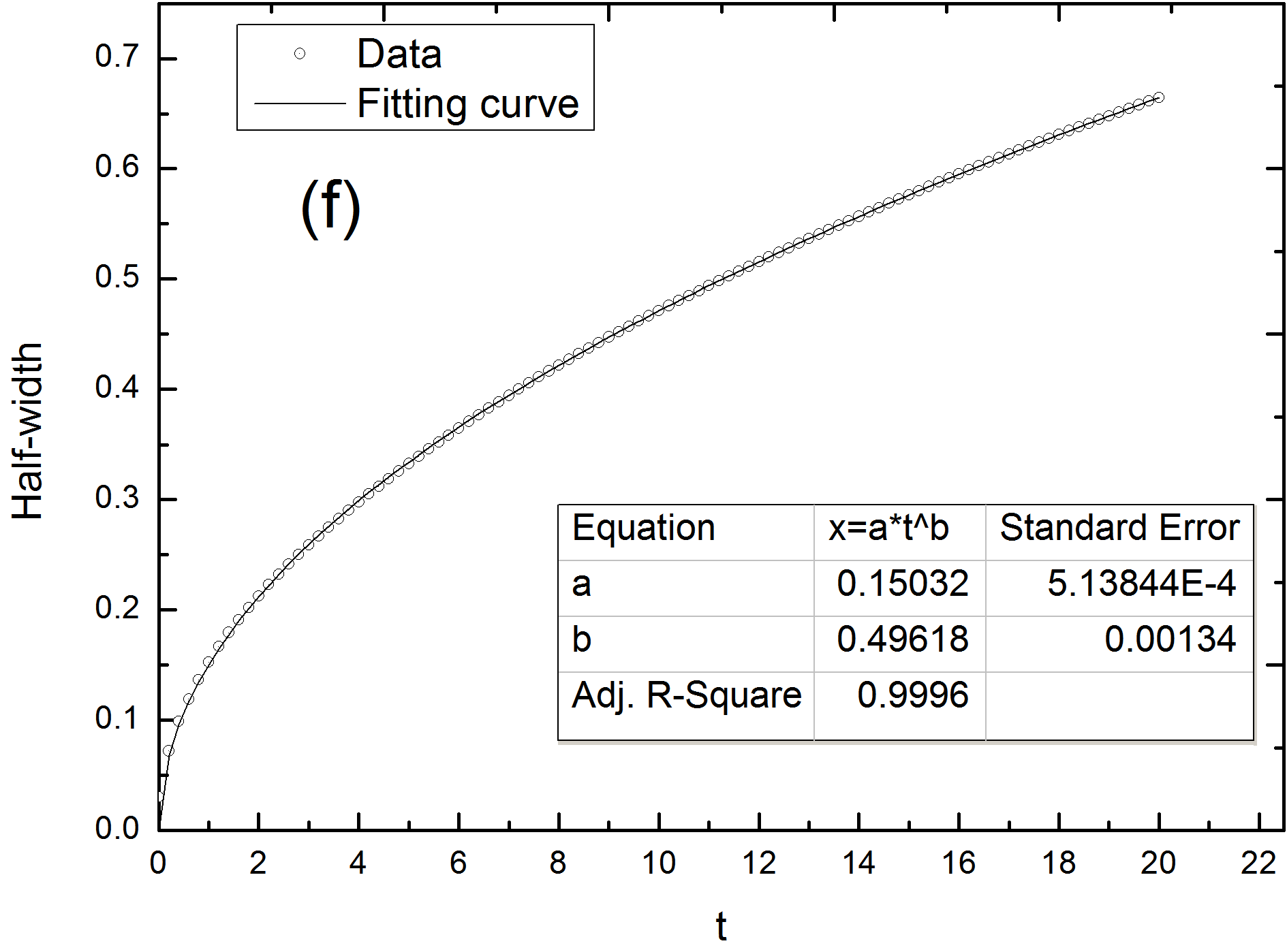


P2: *γ_f_=0.01, γ_G_=λ_a_=λ=β=0*


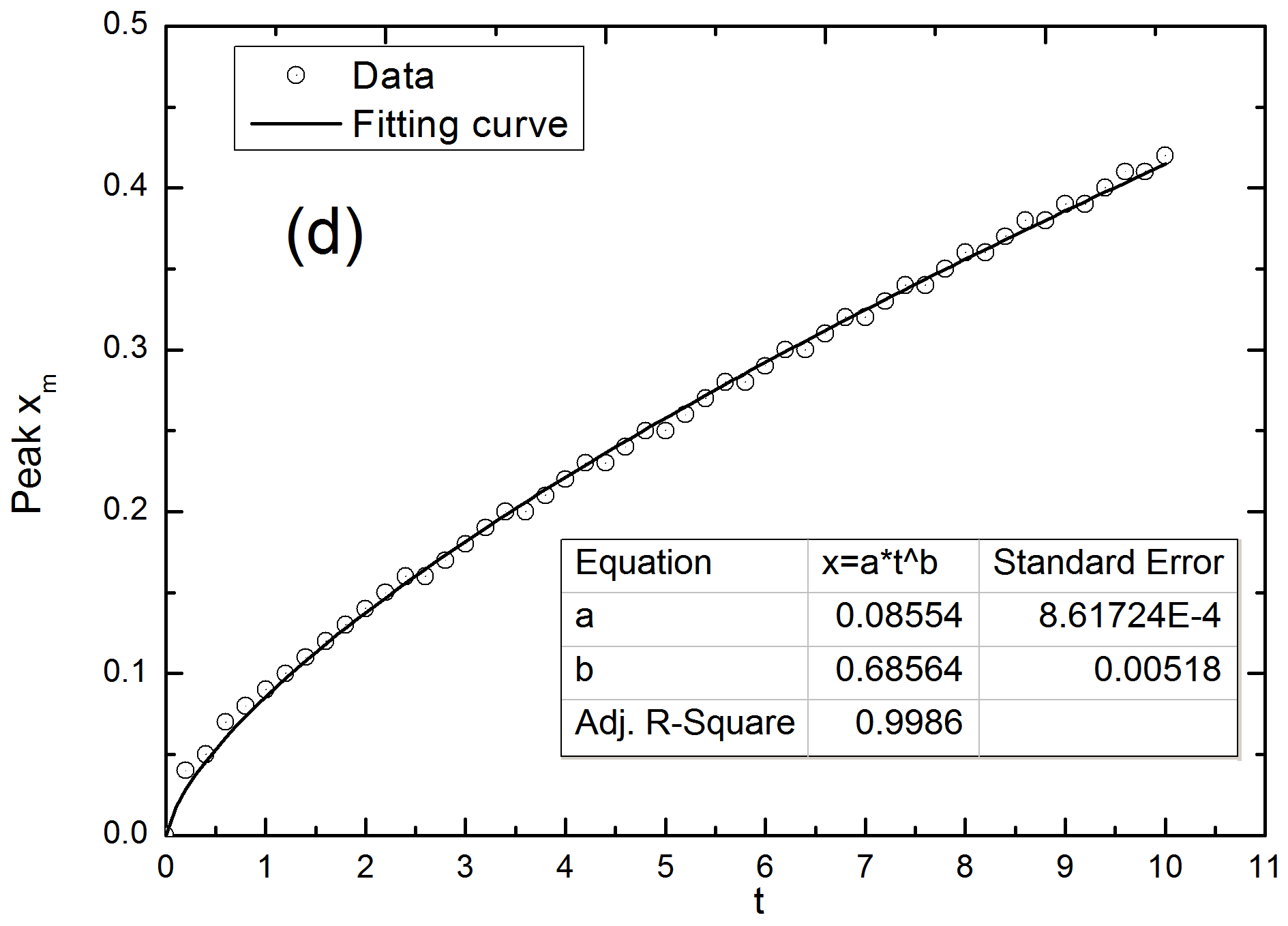


P3: *γ_f_=0, γ_G_=0.00828, λ_a_=λ=β=0*


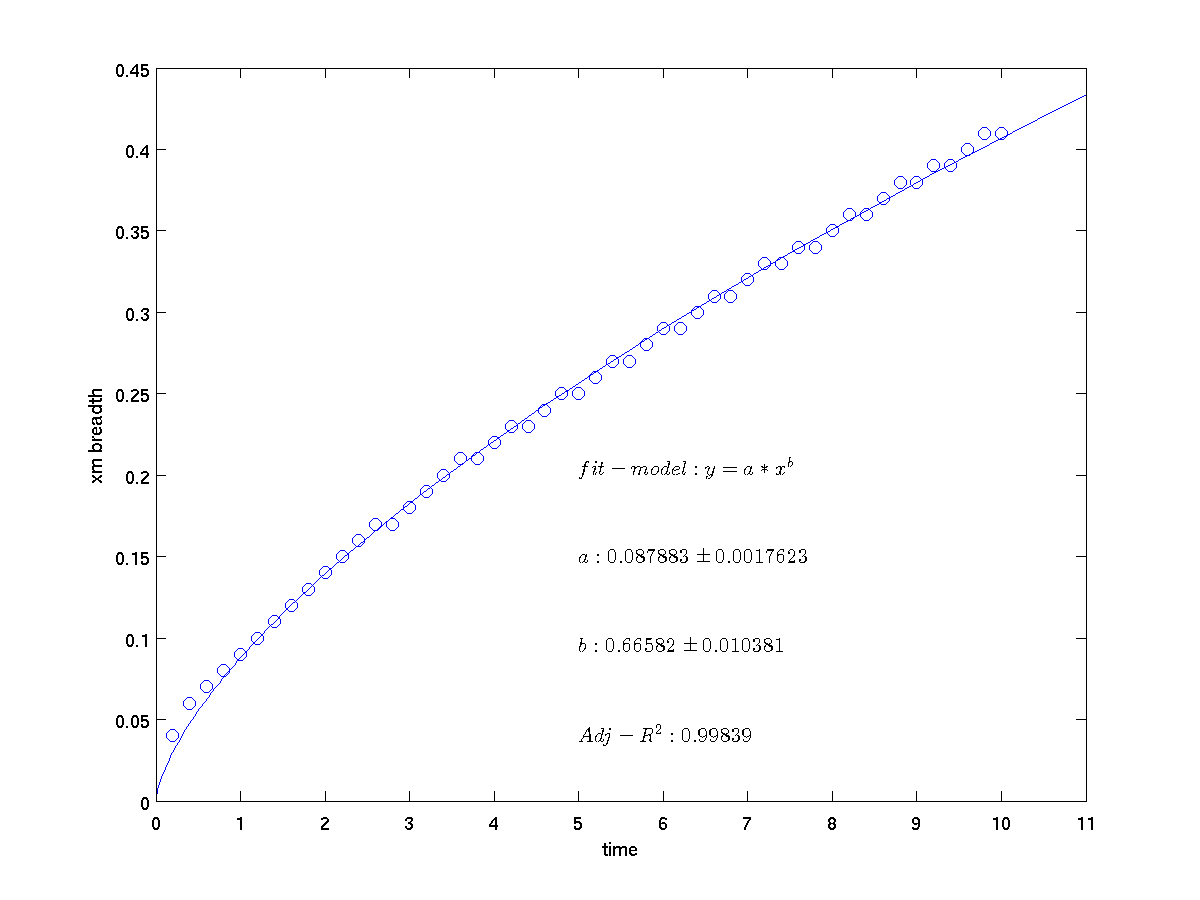


P4: *γ_f_=γ_G_=0, λ_a_=0.003, λ=β=0*


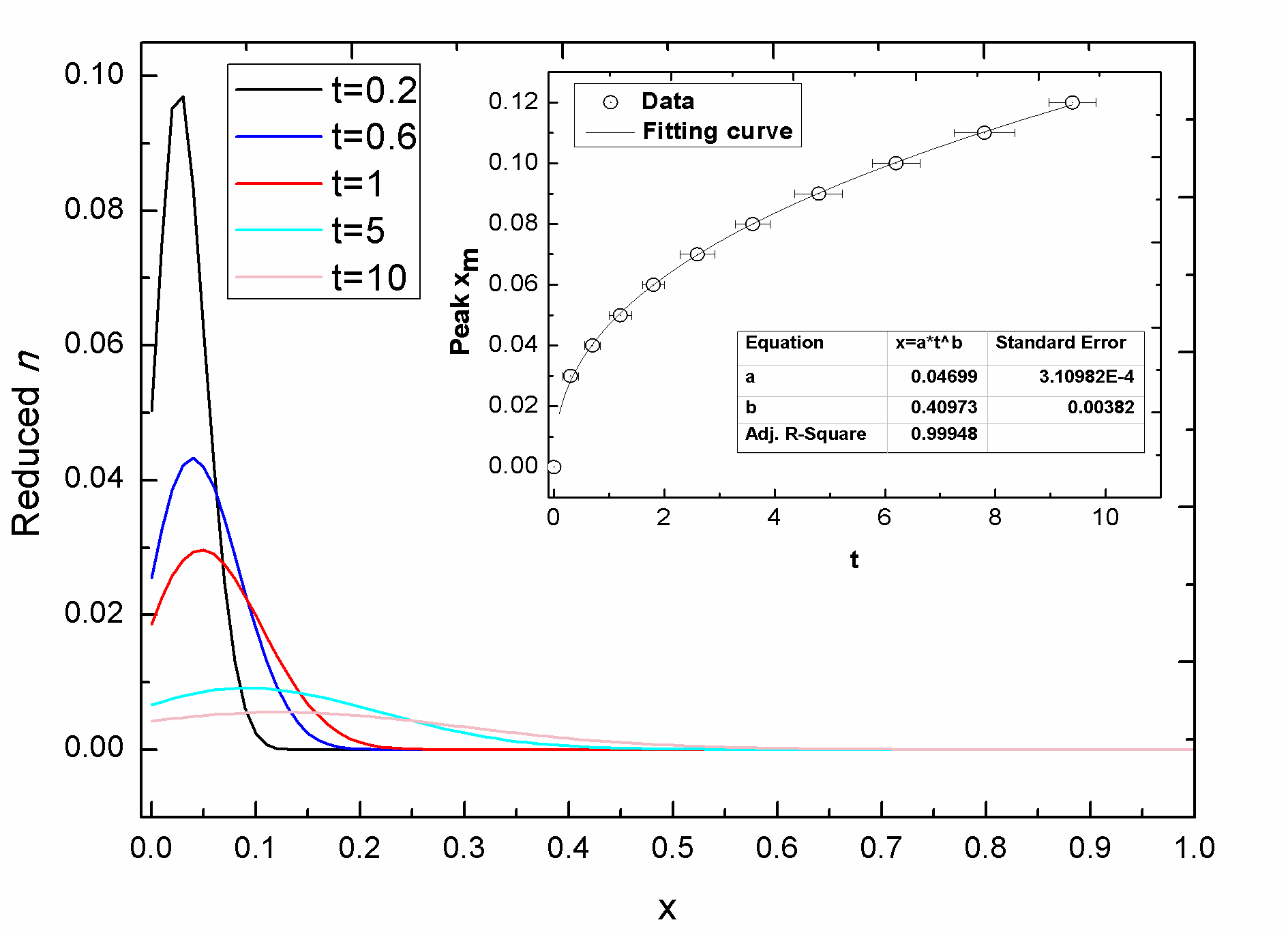


P5: *γ_f_=0, γ_G_=0.00828, λ_a_=0.003, λ=β=0*

*
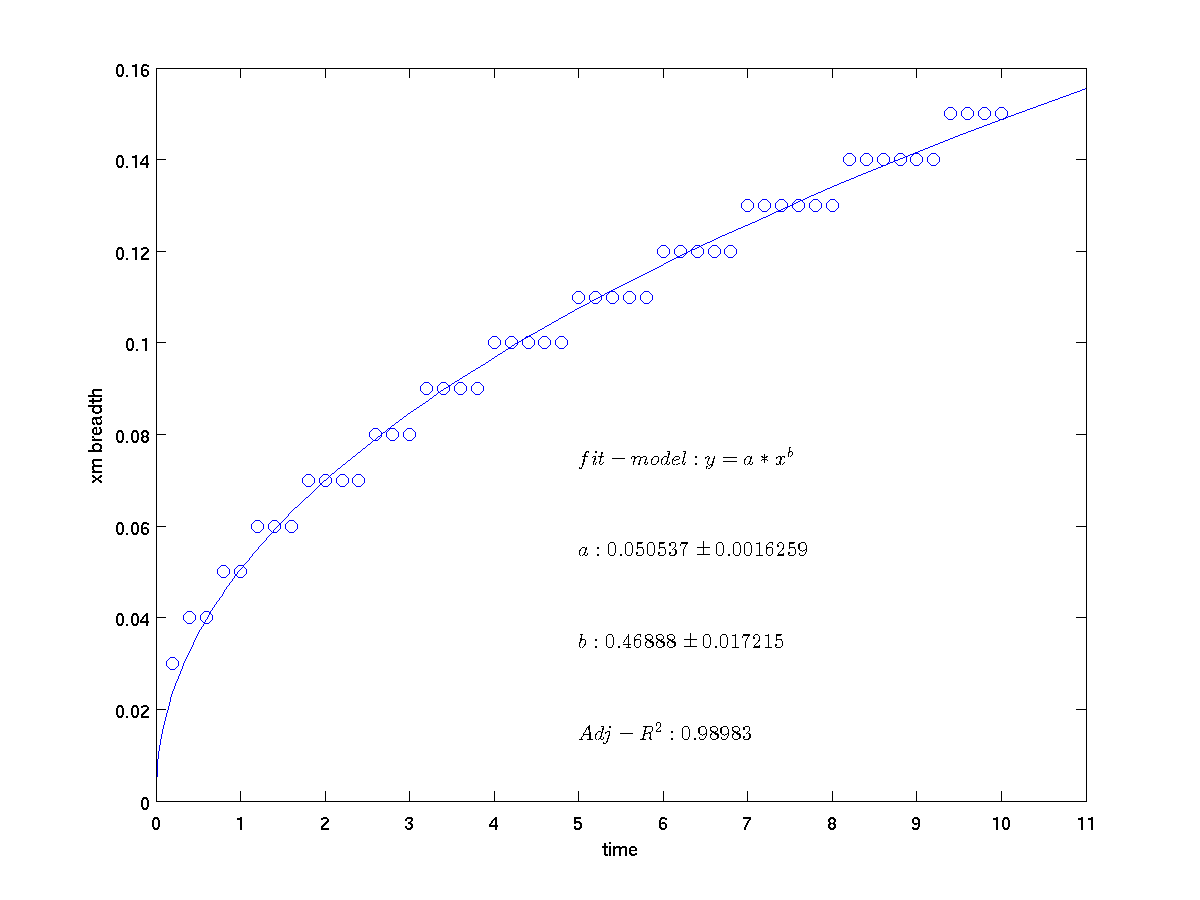
*

P6: *γ_f_=0.01, γ_G_=0, λ_a_=0.003, λ=β=0*

*
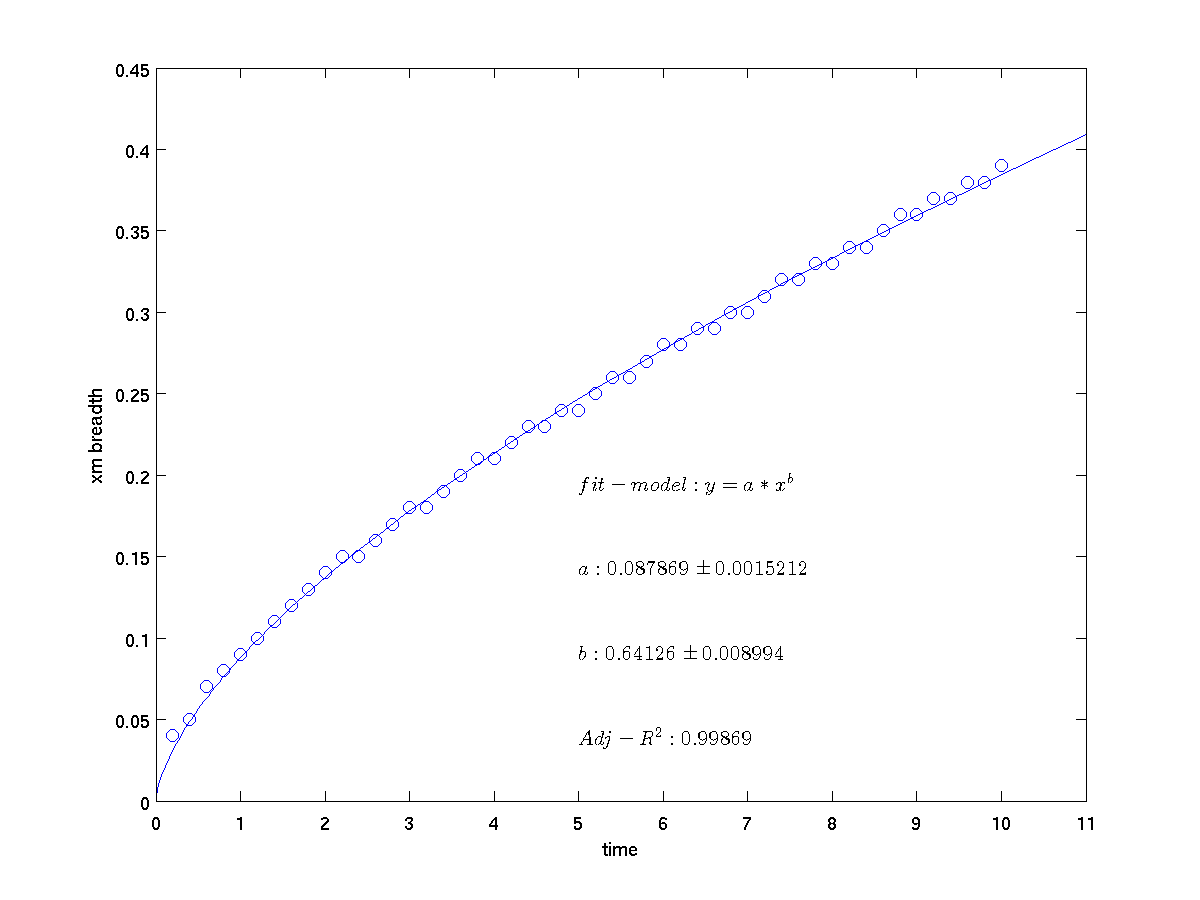
*

P7: *γ_f_=0.01, γ_G_=0.00828, λ_a_=λ=β=0*


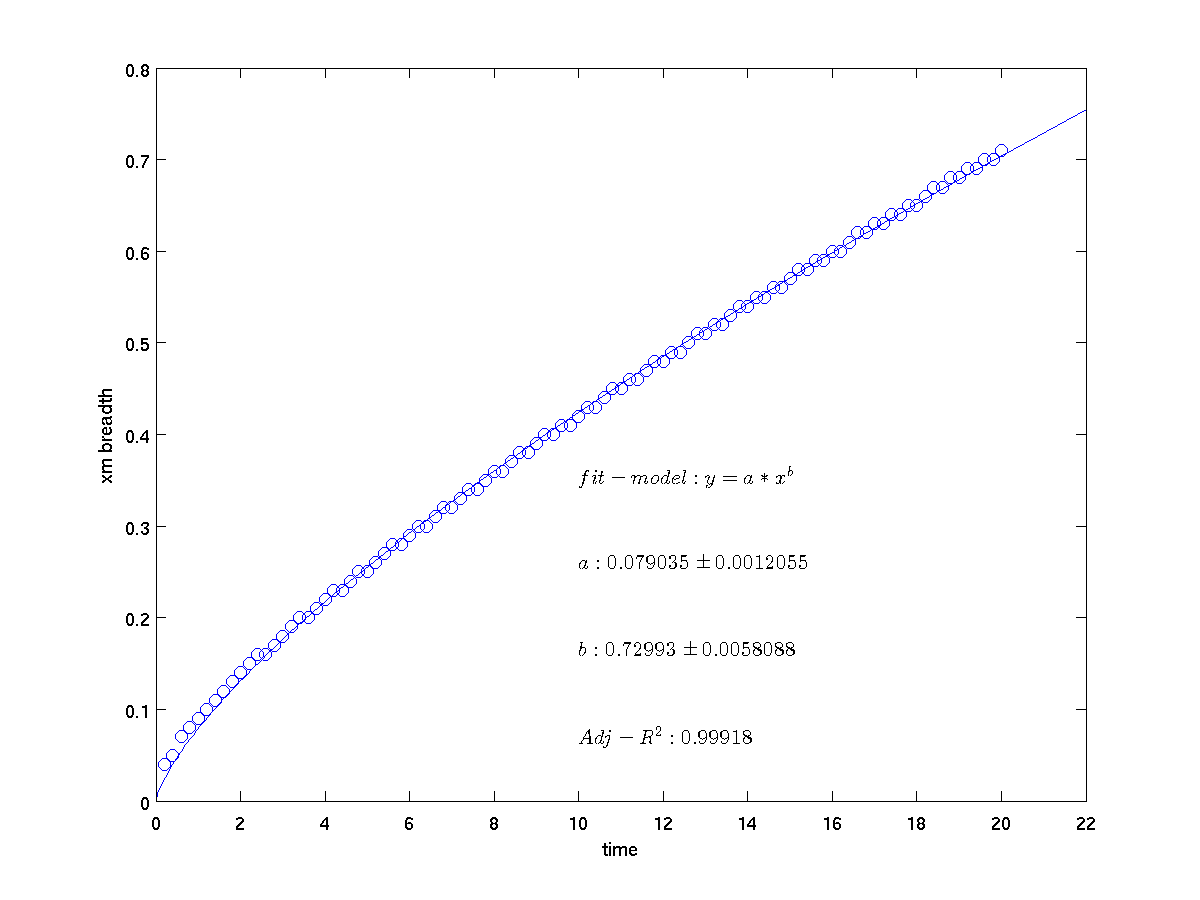


P8: *γ_f_=0.01, γ_G_=0.00828, λ_a_=0.003, λ=β=0*

*
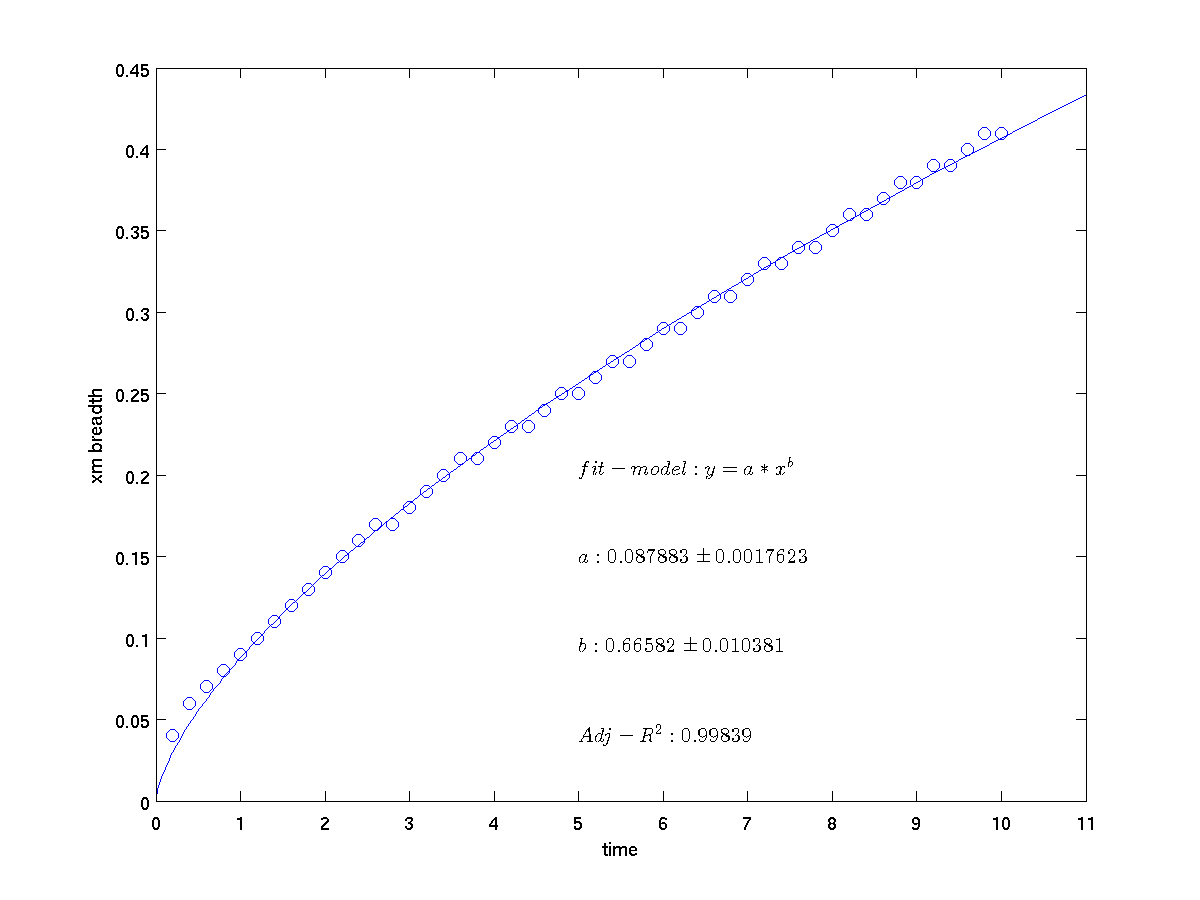
*

P9: *γ_f_=0.01, γ_G_=0.00828, λ_a_=0, λ=1.0, β=2.2*

*
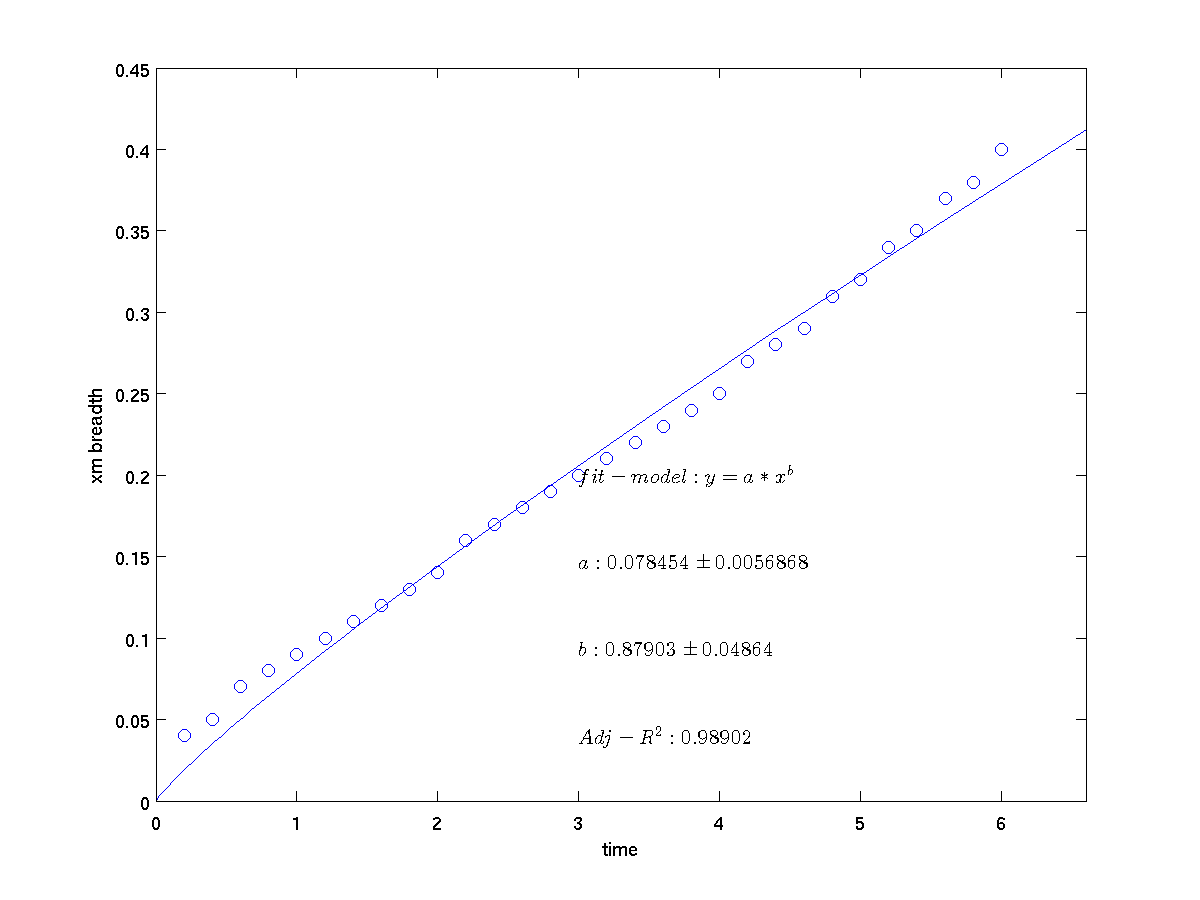
*

P10: *γ_f_=0.01, γ_G_=0.00828, λ_a_=0.003, λ=1.0, β=2.2*

*
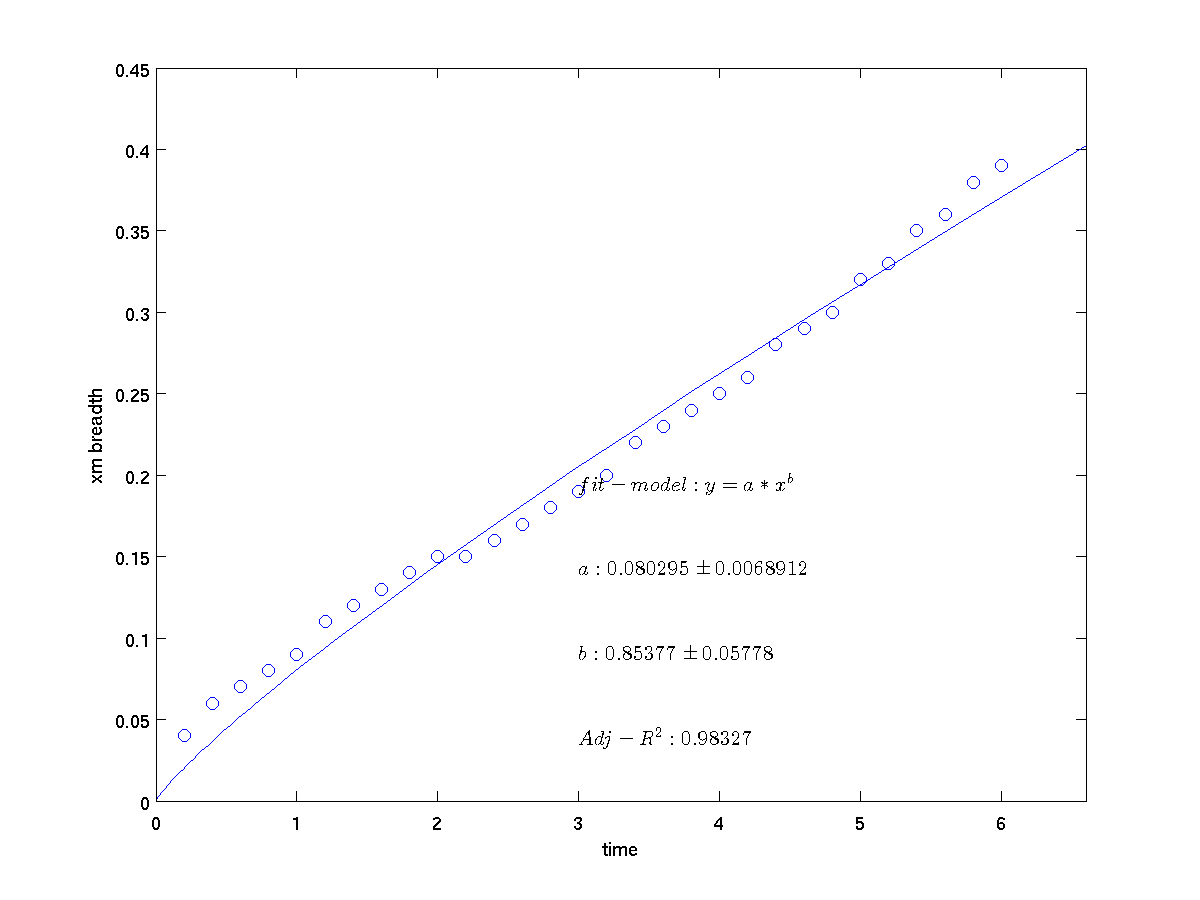
*

P11: *γ_f_=0.01, γ_G_=0.00828, λ_a_=0.003, λ=0.5, β=2.2*


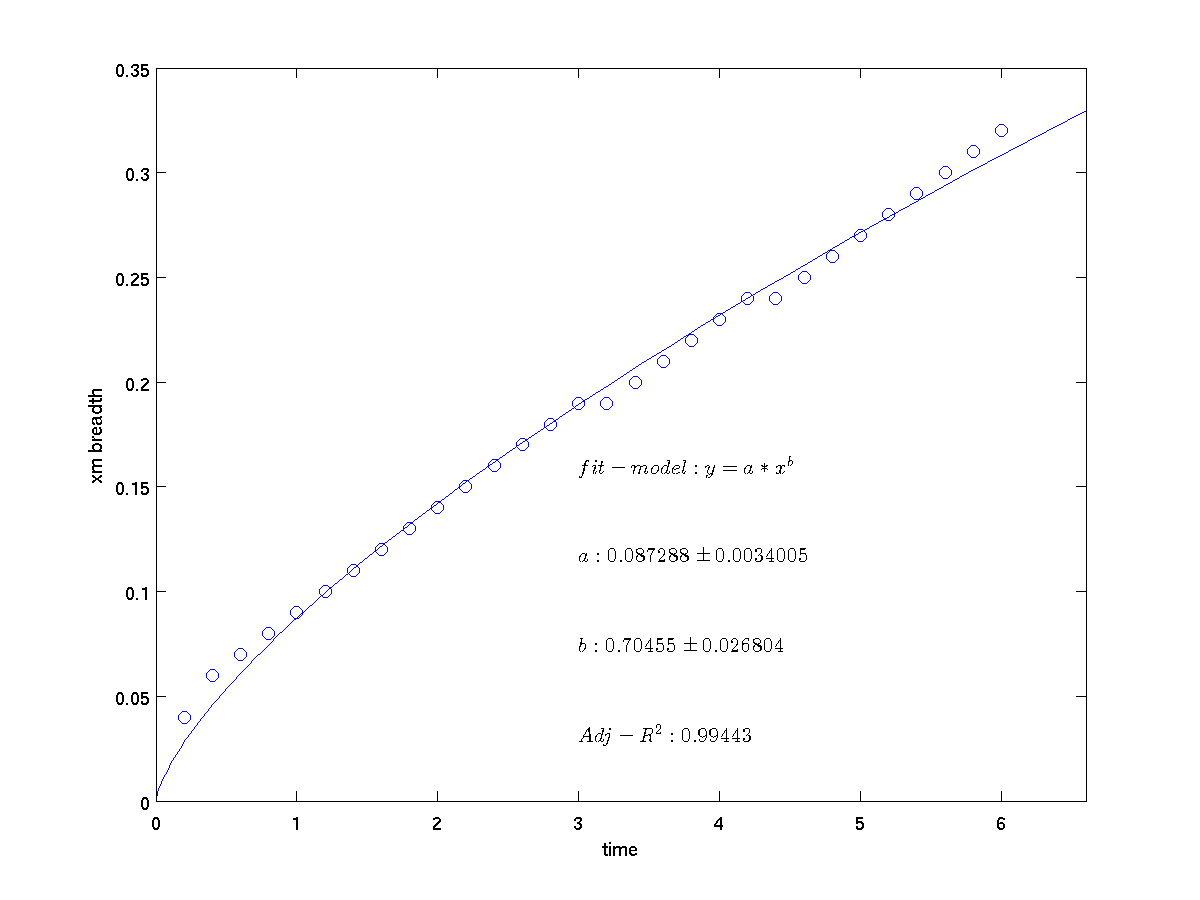


P12: *γ_f_=0.01, γ_G_=0.00828, λ_a_=0.003, λ=1.0, β=4.4*


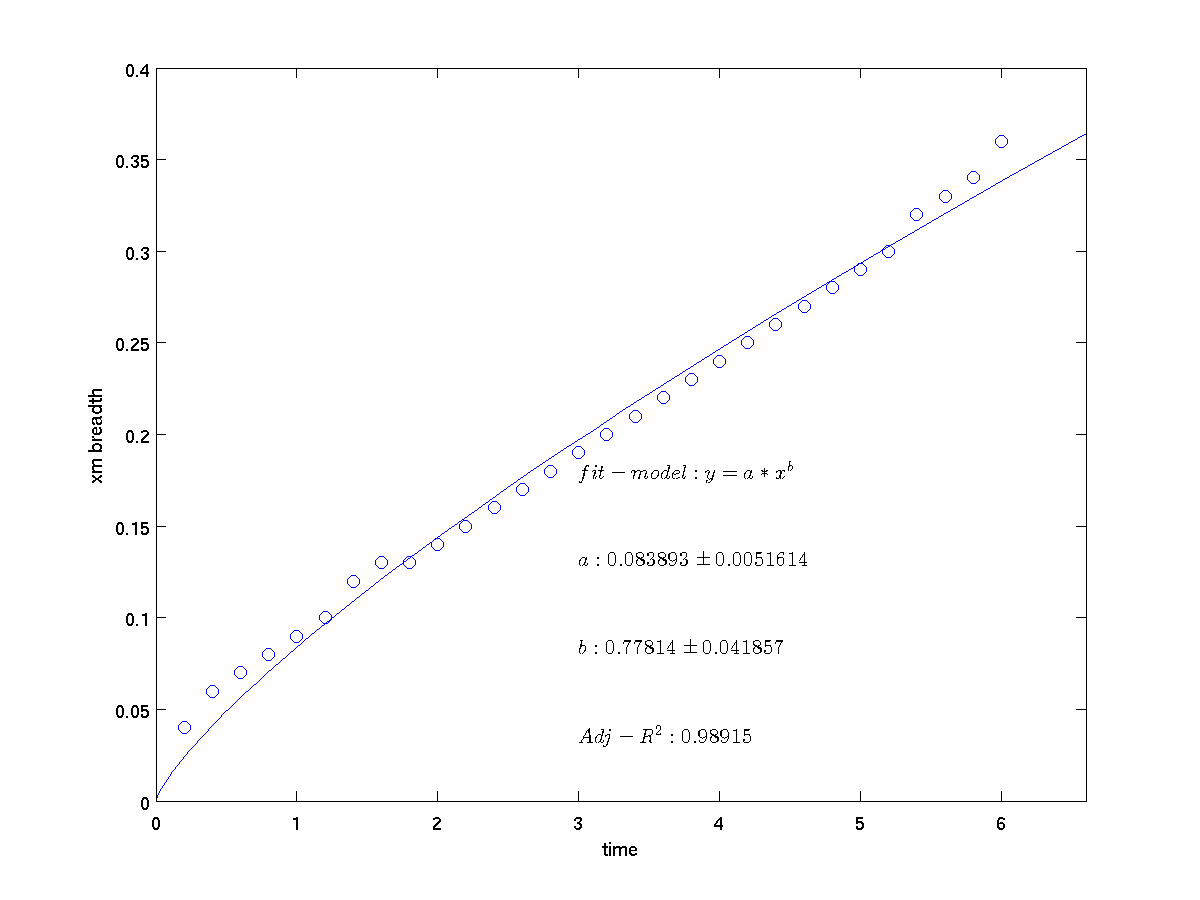


P13: *γ_f_=0.08, γ_G_=0.06, λ_a_=0, λ=2.8, β=1.1*


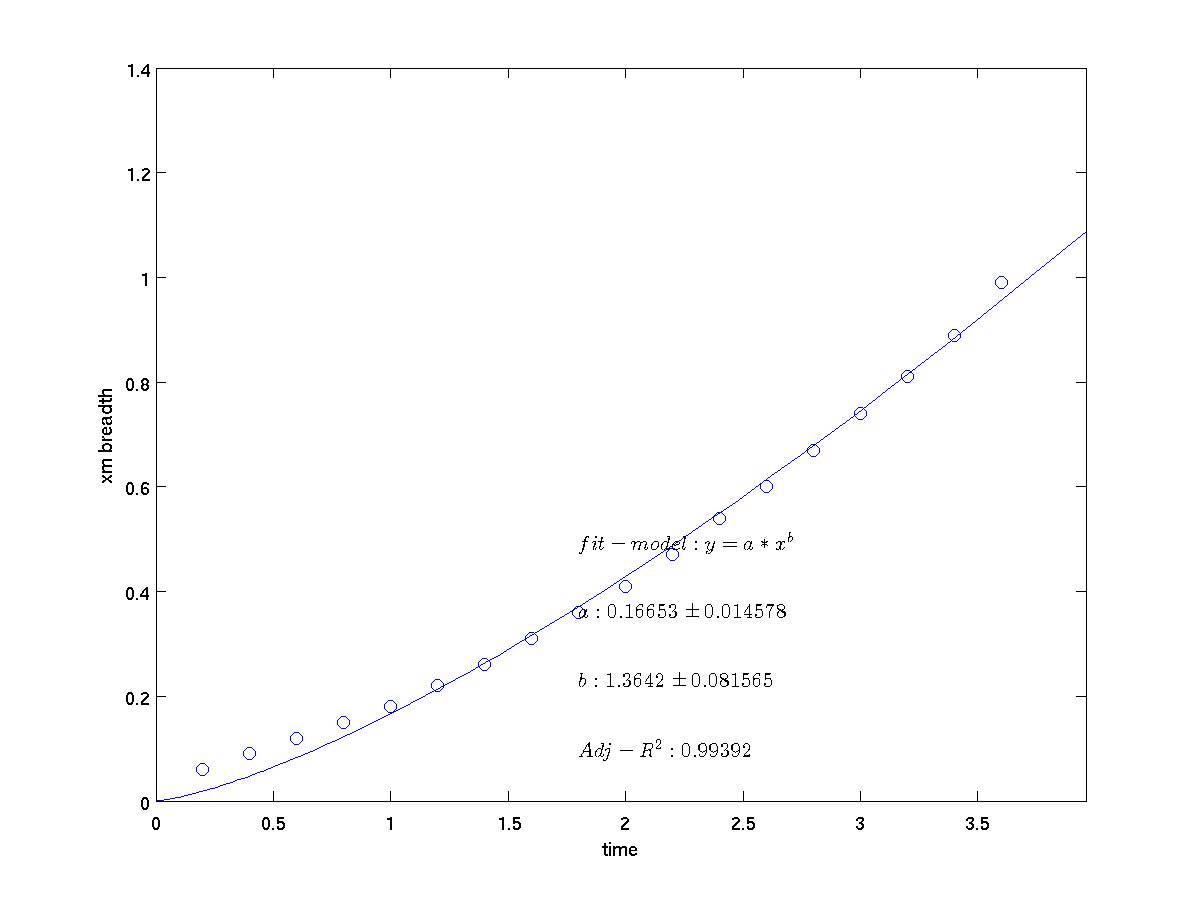


P14: *γ_f_=0.08, γ_G_=0.06, λ_a_=0.00003, λ=2.8, β=1.1*


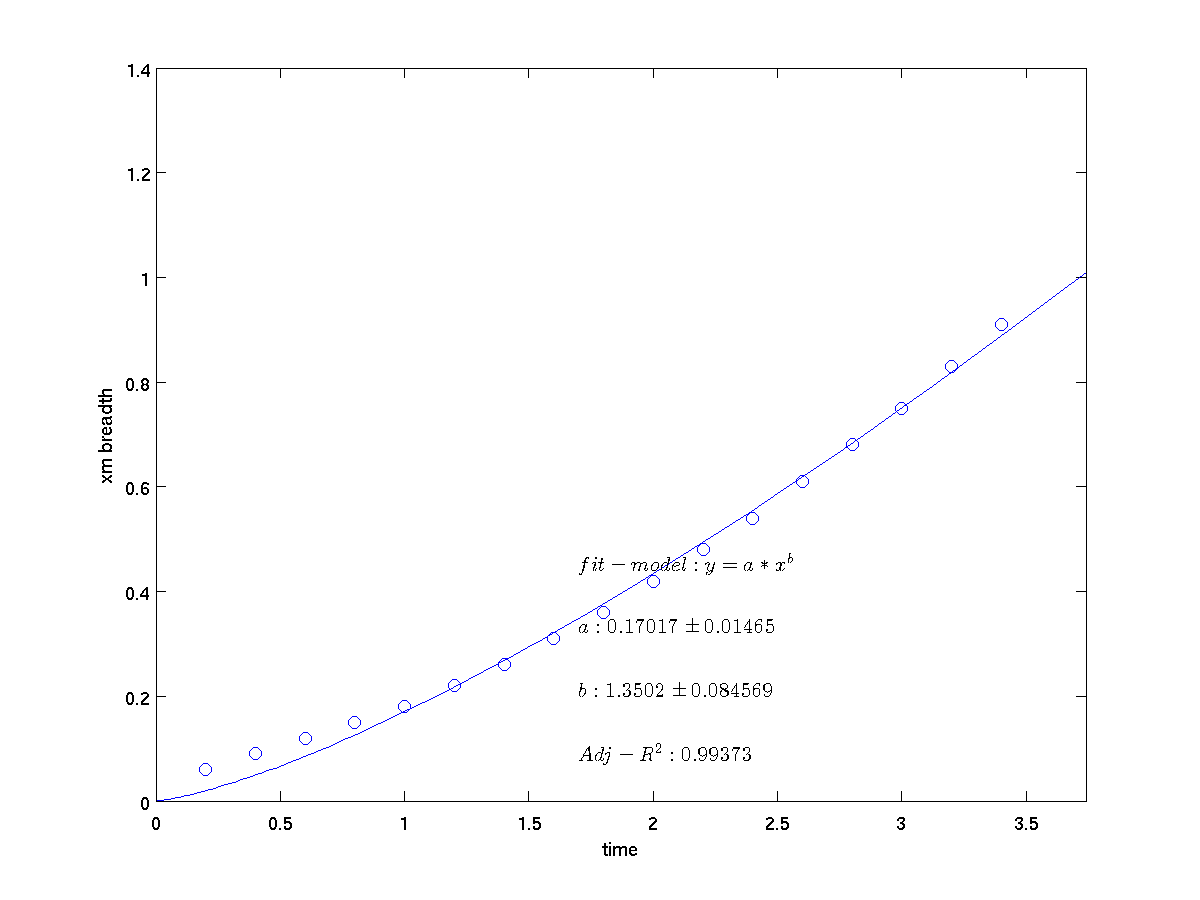

Supplement: File S1 — The simulation results of P1–P14 in Table 3 . (DOC) [file pone.0109784.s002.doc]
