# Supplementary material for: The Anomalous Diffusion of a Tumor Invading with Different Surrounding Tissues
Source: PLoS One. 2014 Oct 13;9(10):e109784. doi: 10.1371/journal.pone.0109784 (PMC4195689; doi:10.1371/journal.pone.0109784)
Supplement: File S2 — The materials and Methods information of cell cultures. (DOC) [file pone.0109784.s003.doc]

**S2 The materials and Methods information of cell culture**

Cell Culture and Generation of Stably Transduced Cell Lines. A549 cell line: 96-well ULA plates are used in cell culture, cells were maintained in cell culture media, supplemented with 10% FBS, 1% Glutamax, 1% Pen/Strep, 0.5 μg ml^-1^ Puromycin at 37℃ in 5% CO_2_ (Essen BioScience, Inc, Catalog Number: 4491) for more details. SiHa human cervical carcinoma cell line: 96-well ULA plates are used in cell culture, cells were cultured in complete MEM medium (Mediatech Inc, Manassas, VA) with 10% FBS and 1% antibiotic/antimicrobial at 37℃ in 5% CO_2_. The more details can be found in Kim's work [1]. MDA-MB-231cell line: 96-well ULA plates are used in cell culture, cells were maintained in DMEM 10 µg/ml gentamycin at 37℃ in 5% CO_2_ (Trevigen Inc, Cultrex Catalog #: 3500-096-K). U-87 MG cell line: two different kind of plates, ultra-low attachment (ULA) and agar plates are used in cell culture respectively; cells were maintained in DMEM (4.5 g/l D-glucose; sodium pyruvate), supplemented with 15% FCS, 1 × penicillin, streptomycin and neomycin antibiotic mix (Gibco, Life Technologies Ltd. Paysley, UK), 100μMb-mercaptoethanol (Sigma-Aldrich Company Ltd., Dorset, England), 1 × NEAA plus 1,000 U/ml leukemia inhibitor factor (LIF) at 37℃ in 5% CO_2_. The more details can be found in Vinci's work [2].

References

1. Kim T-H, Mount CW, Gombotz WR, Pun SH (2010) The delivery of doxorubicin to 3-D multicellular spheroids and tumors in a murine xenograft model using tumor-penetrating triblock polymeric micelles. Biomaterials 31: 7386–7397.

2. Vinci M, Gowan S, Boxall F, Patterson L, Zimmermann M, et al. (2012) Advances in establishment and analysis of three-dimensional tumor spheroid-based functional assays for target validation and drug evaluation. BMC Biol 10: 29.
