# Supplementary material for: The Anomalous Diffusion of a Tumor Invading with Different Surrounding Tissues
Source: PLoS One. 2014 Oct 13;9(10):e109784. doi: 10.1371/journal.pone.0109784 (PMC4195689; doi:10.1371/journal.pone.0109784)

**S3** **The data and curve fitting of A549 cell line, SiHa HCC cell line, U87 MG cell line (ULA), and U87 MG cell line (agar)**

The data and curve fitting of A549 cell line

t=0h
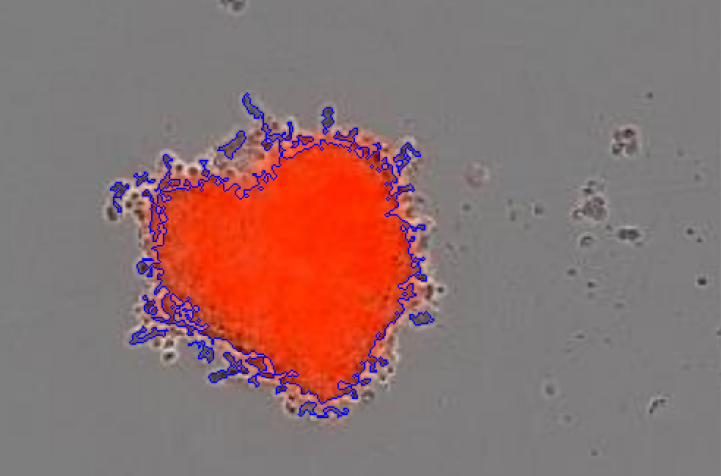


t=96h
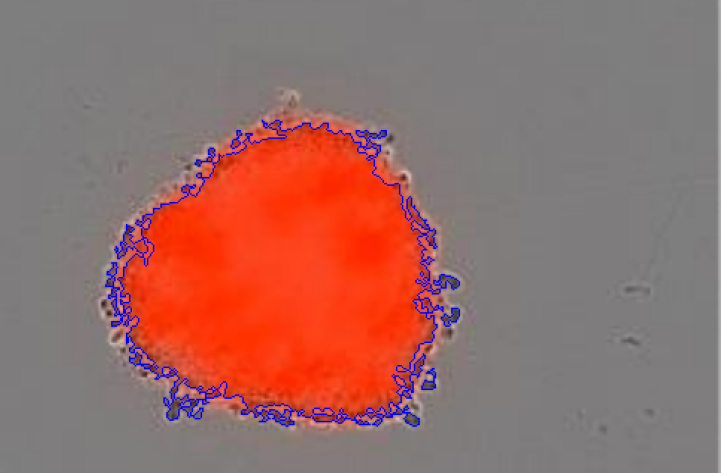


t=198h
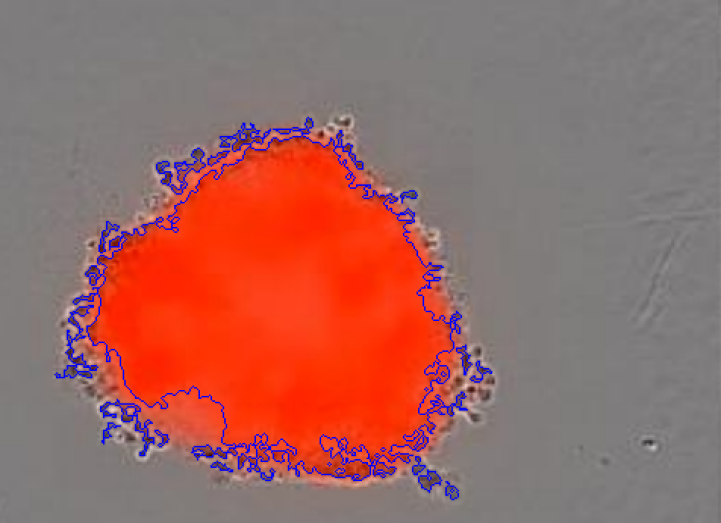


t=258h
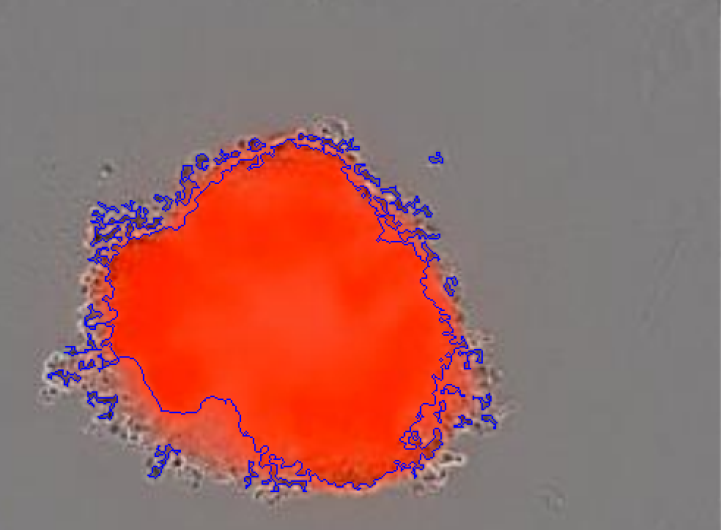


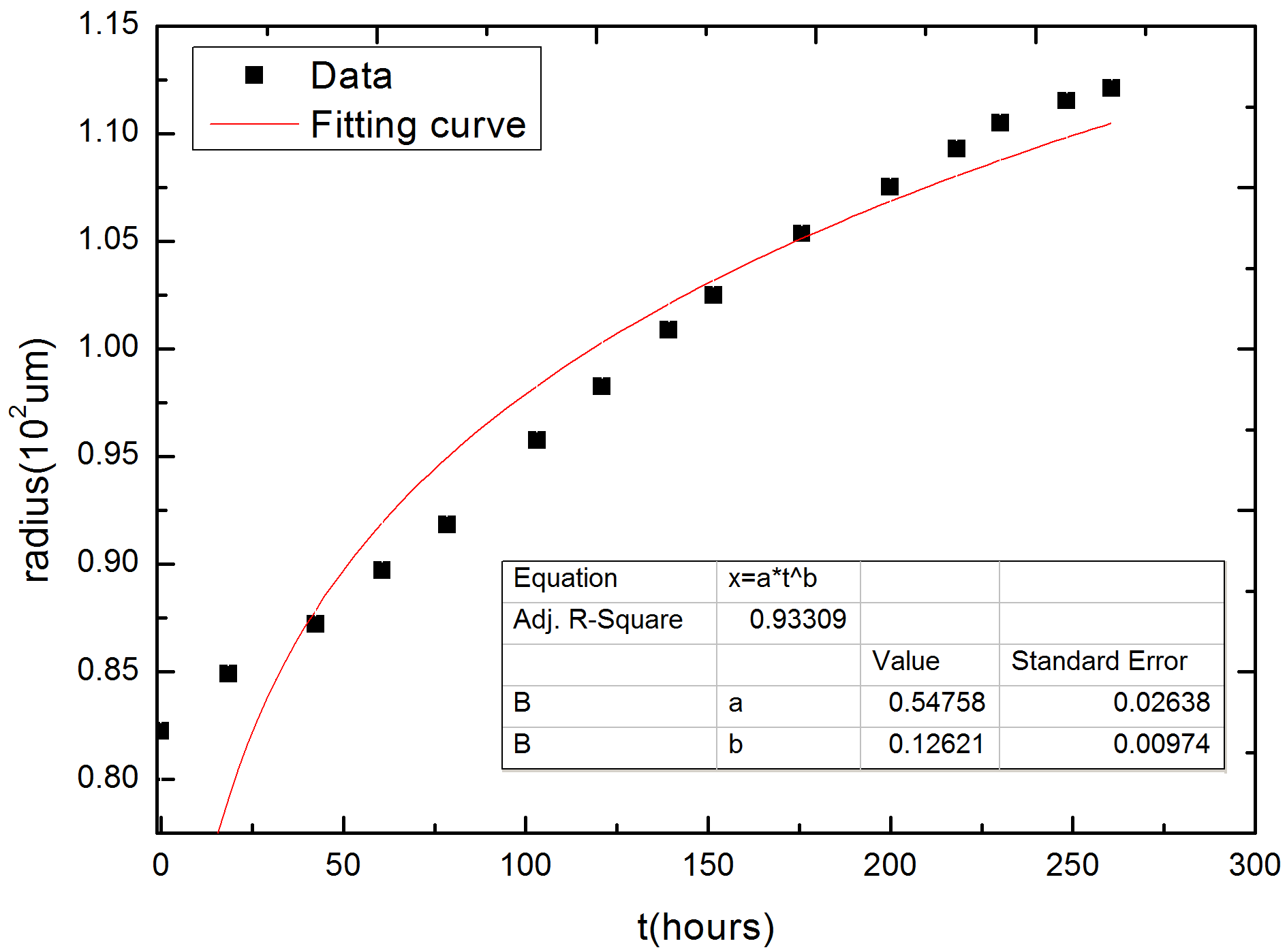


The data and curve fitting of SiHa HCC cell line


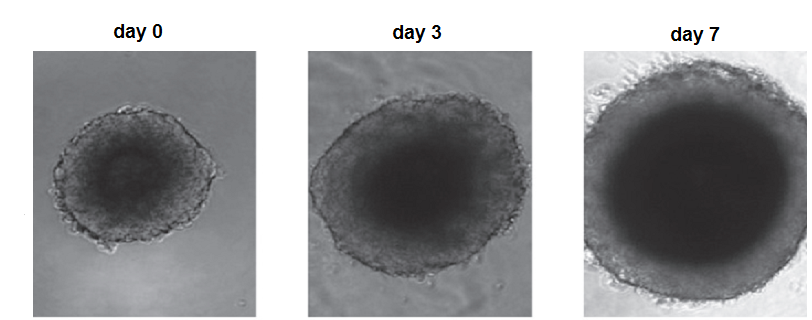


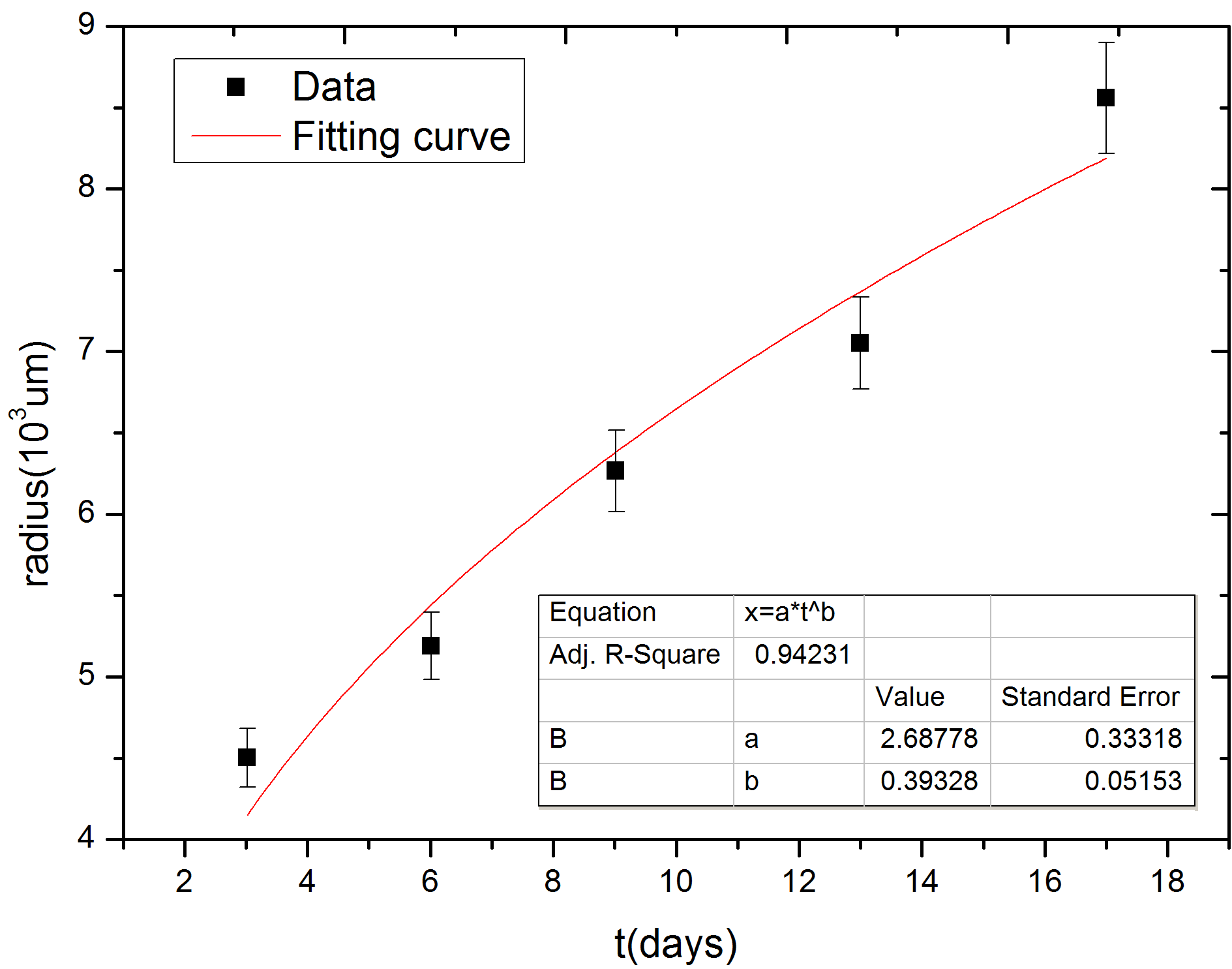


The data and curve fitting of U87 MG cell line


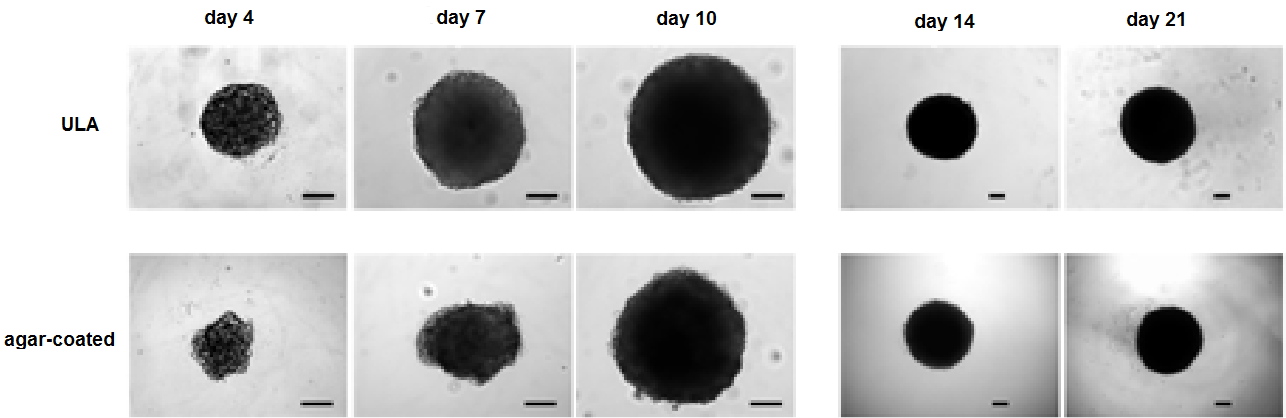


The curve fitting of U87 MG cell line (ULA)


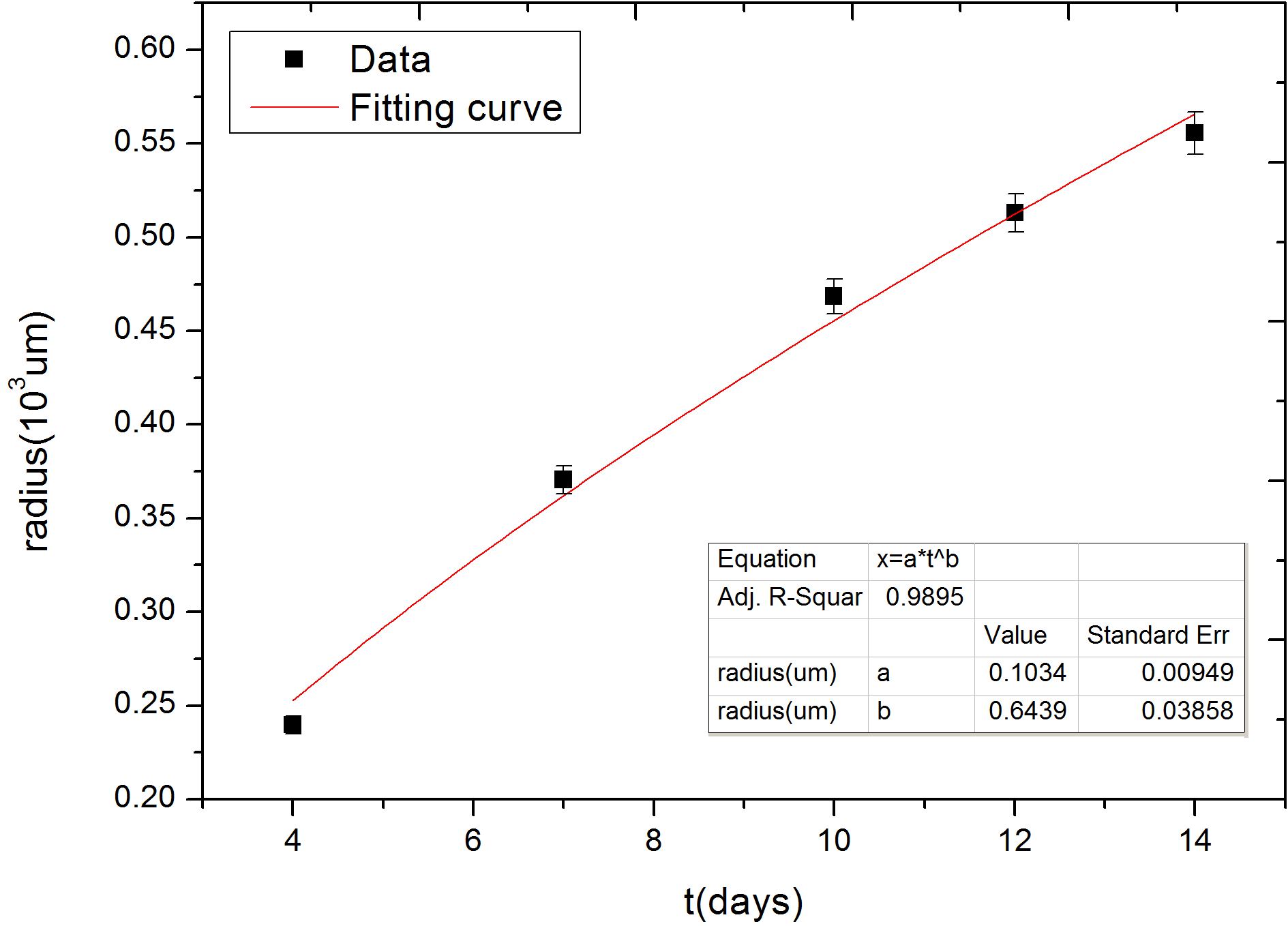


The curve fitting of U87 MG cell line (agar-coated)


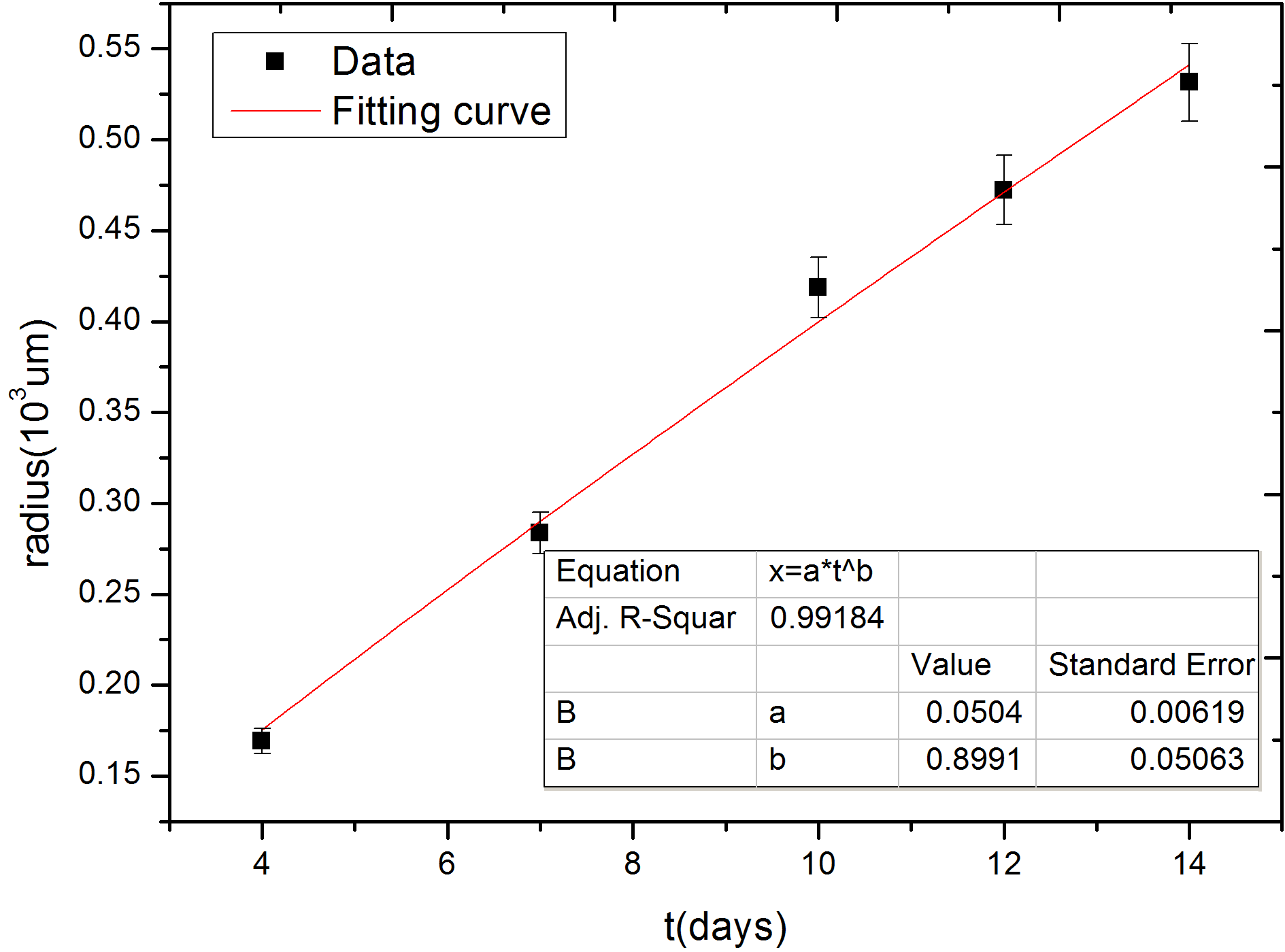

Supplement: File S3 — The data and curve fitting of A549 cell line, SiHa HCC cell line, U87 MG cell line (ULA), and U87 MG cell line (agar). The A549 cell line was cultured without matrix, and the original images can be found in Essen BioScience Inc, Catalog Number: 4491 [1]; the SiHa HCC cell line was also cultured without matrix. Please refer to Kim's work [2] for the original data; both of the U87 MG cell lines were cultured with matrix, and the original data can be found in Vinci's work [3]. (DOC) [file pone.0109784.s004.doc]
