# Supplementary material for: The Anomalous Diffusion of a Tumor Invading with Different Surrounding Tissues
Source: PLoS One. 2014 Oct 13;9(10):e109784. doi: 10.1371/journal.pone.0109784 (PMC4195689; doi:10.1371/journal.pone.0109784)

**S4** The Sun Yat-sen University Cancer Center provided data from three cancer patients who had been clinically diagnosed with an adrenal metastatic tumor (patient 1 with an expansive growing tumor) or a liver metastatic tumor (patient 2 with an infiltrative growing tumor, patient 3 with three infiltrative growing tumors). For the sake of convenience, the liver tumors were marked in picture with liver A,B,C and D, respectively.

Patient1: Adrenal tumors


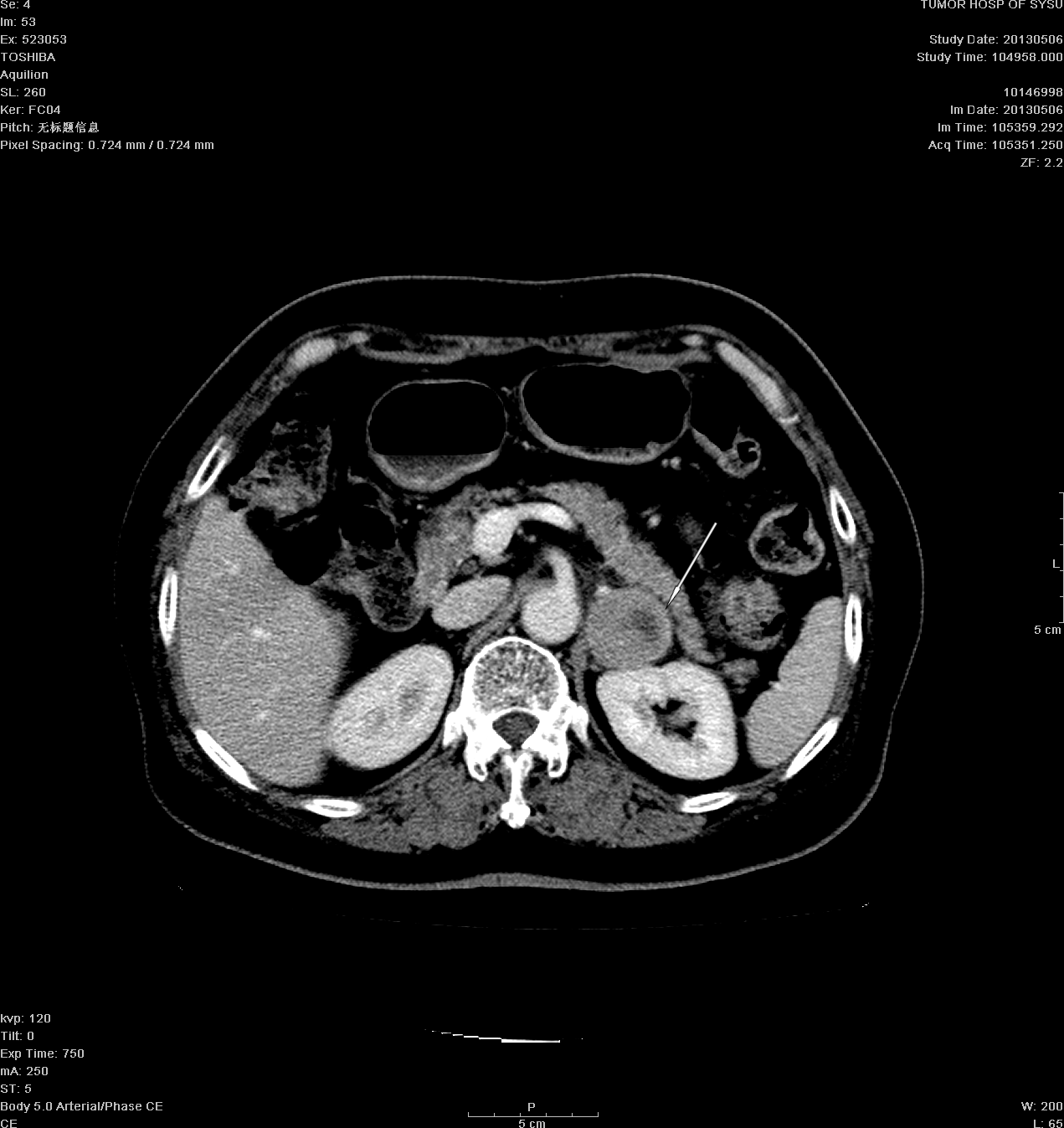


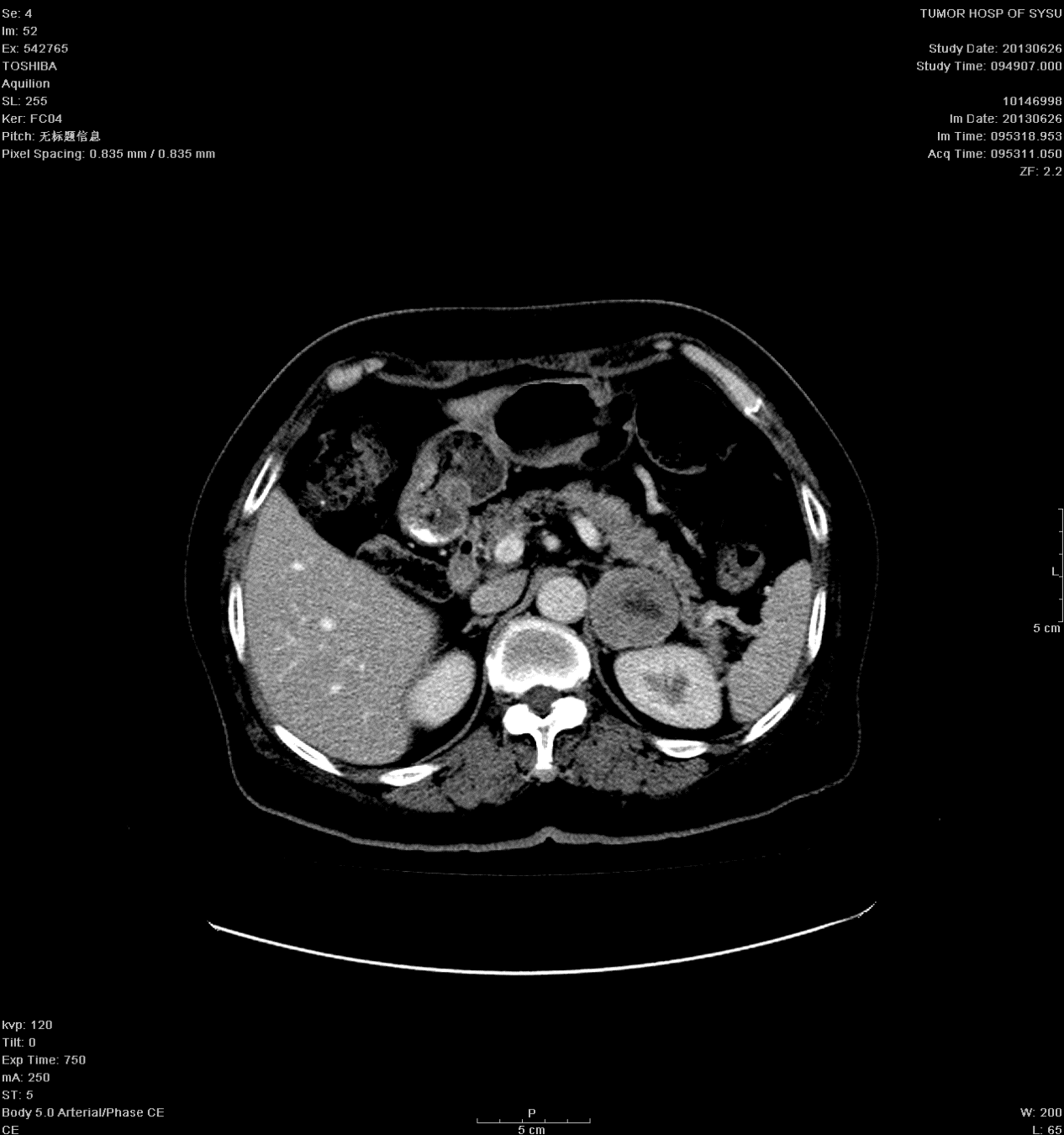


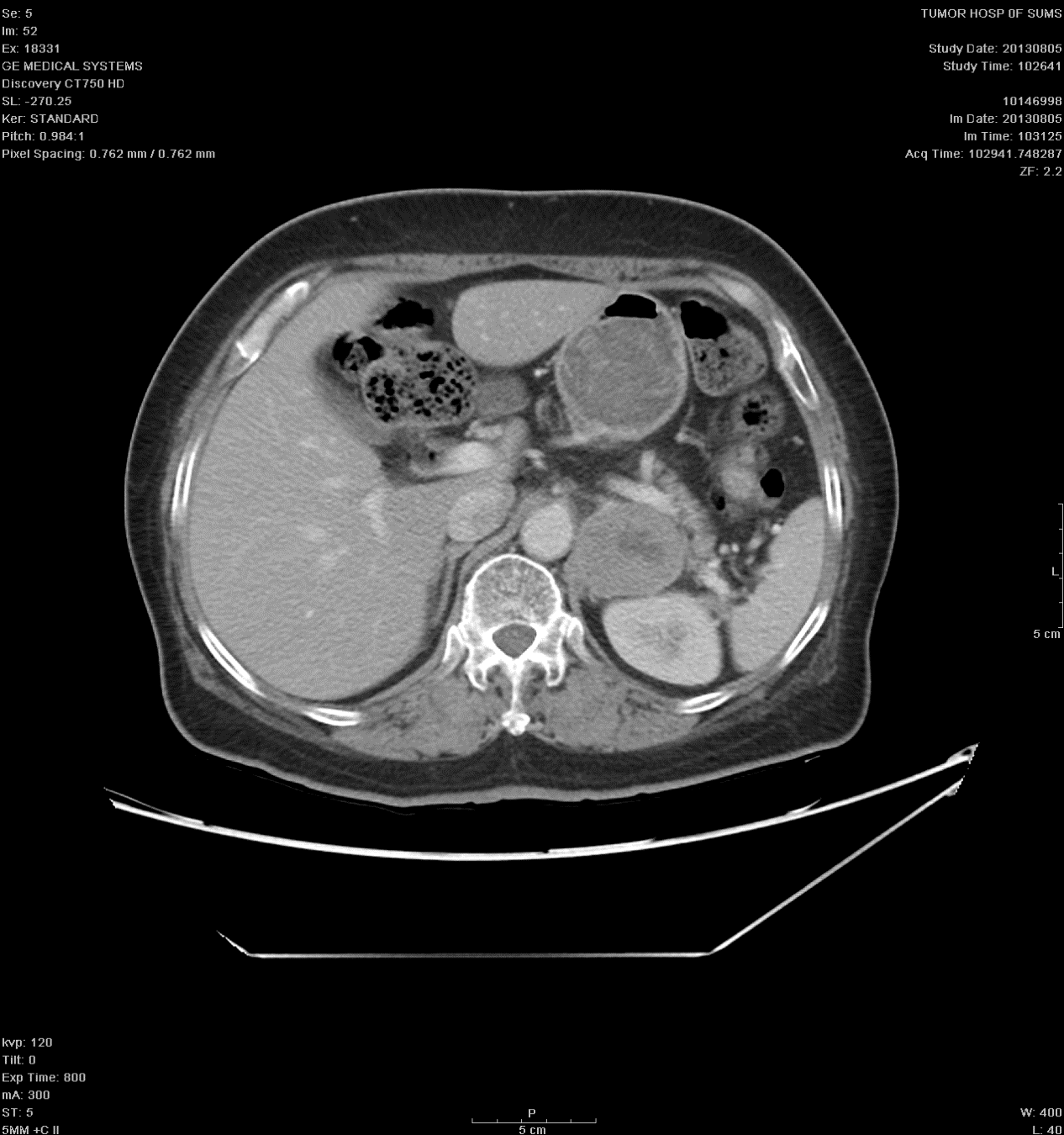


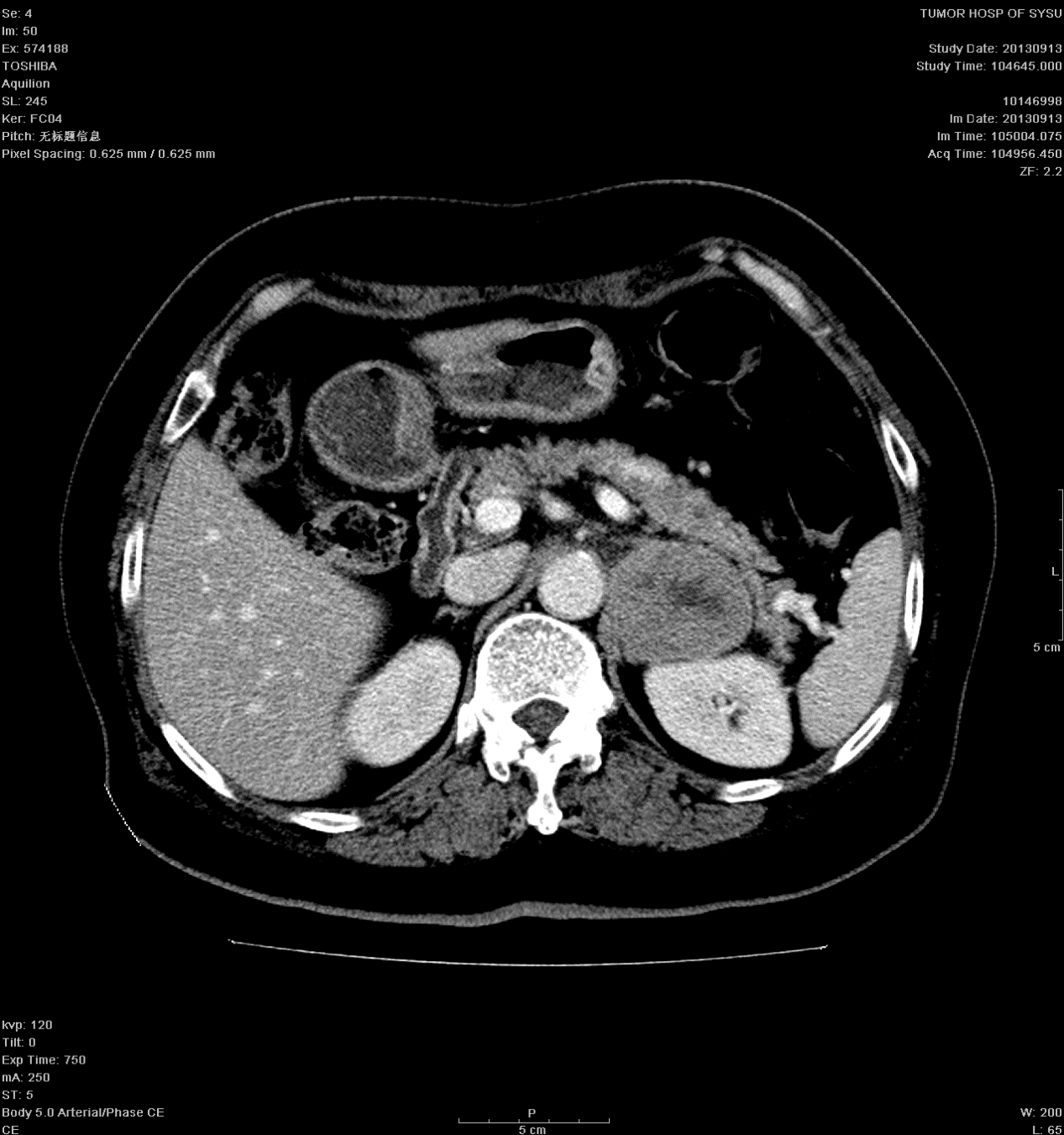


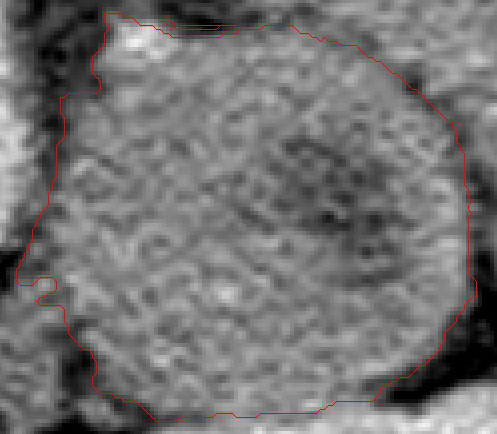


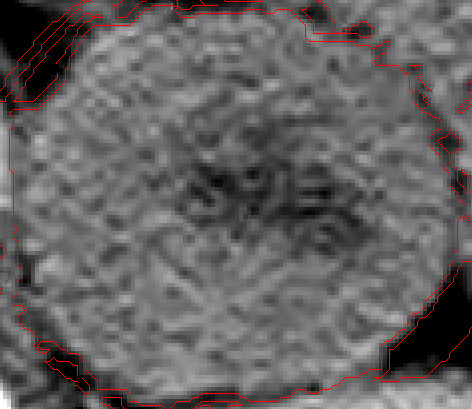


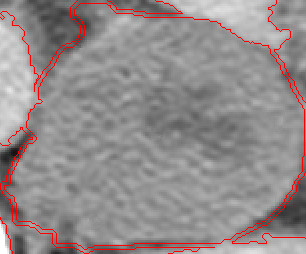


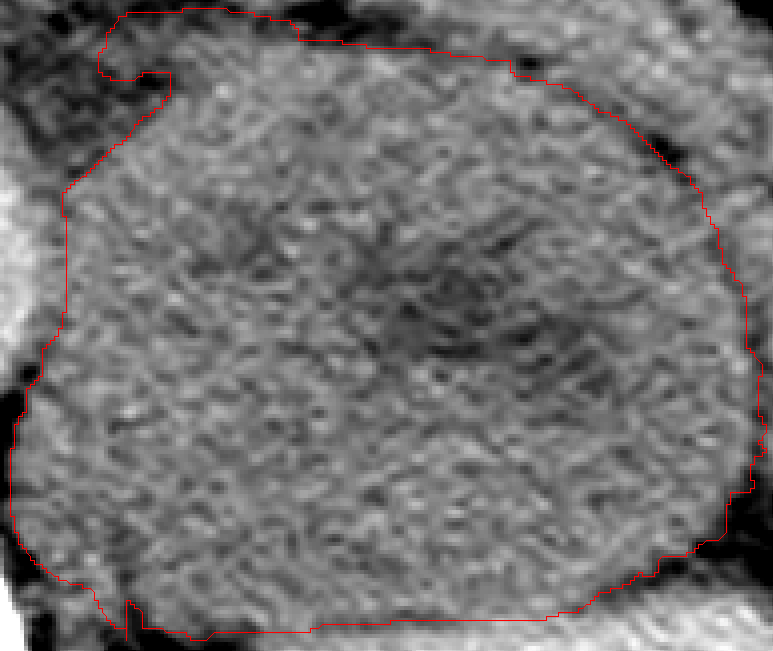


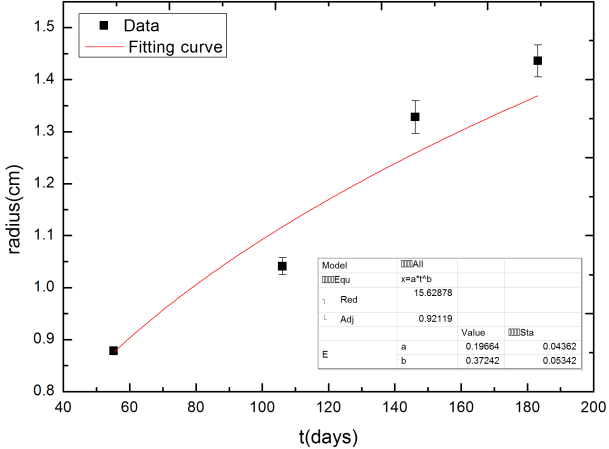


Patient2：Liver tumors A


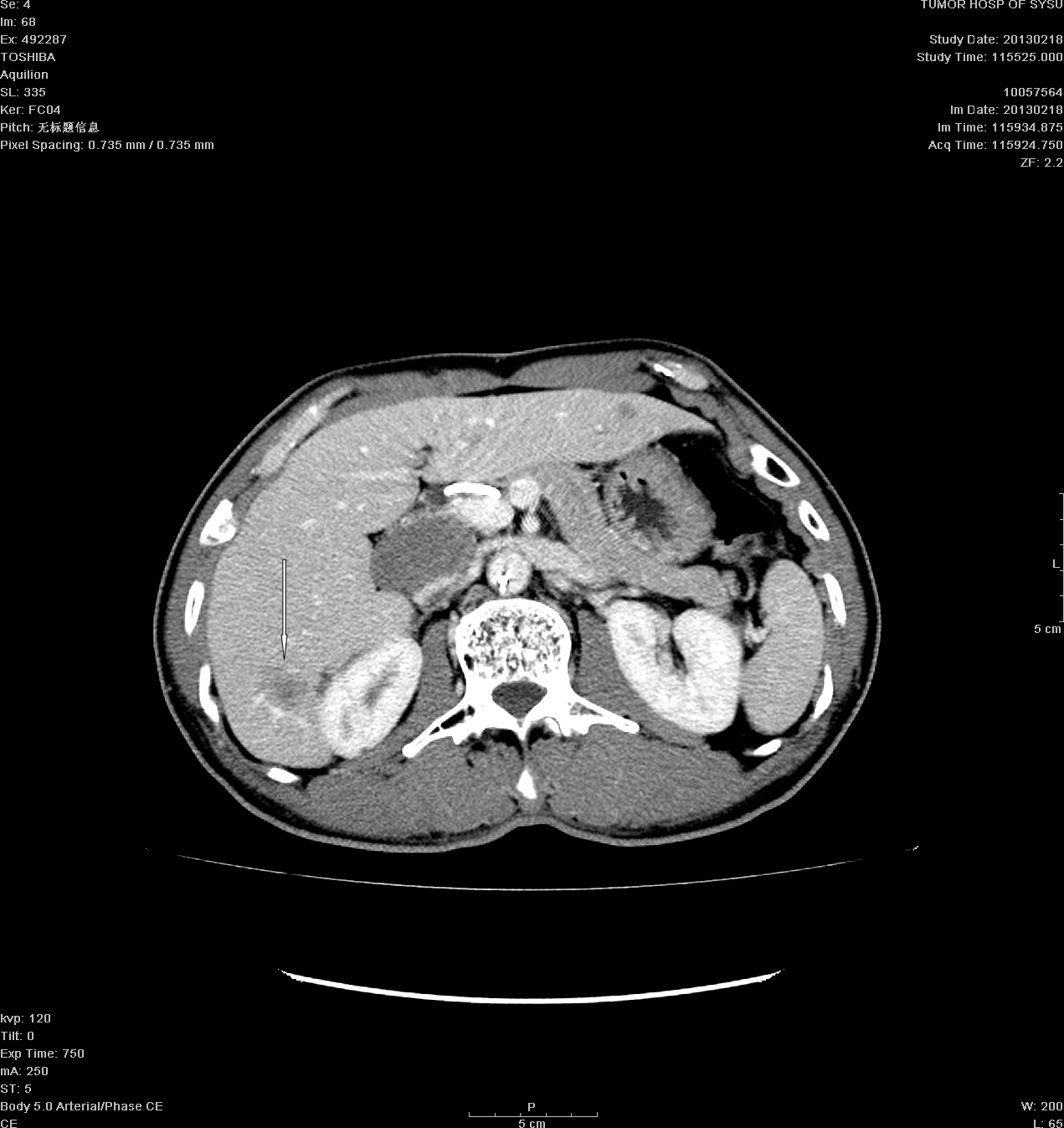


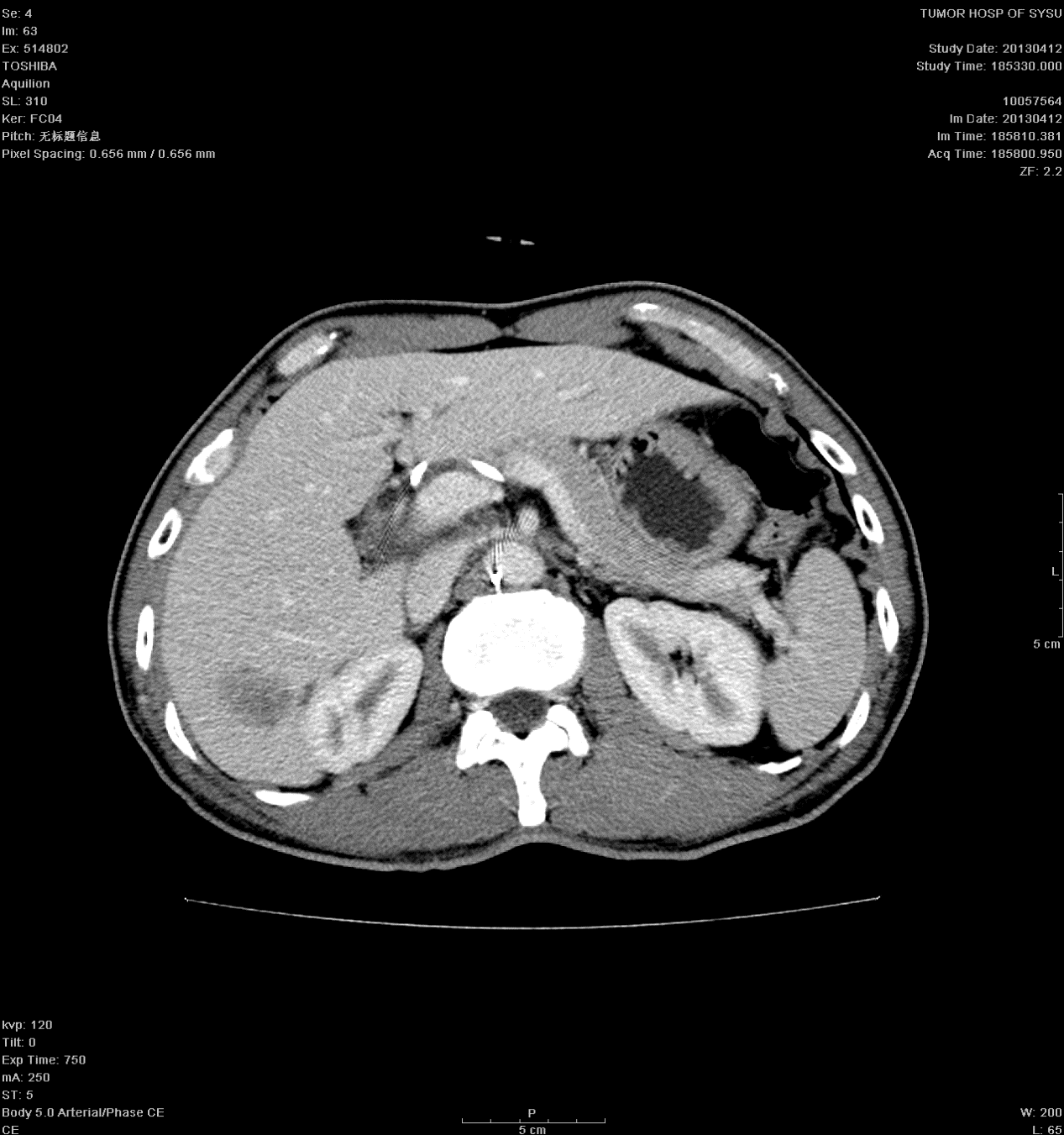


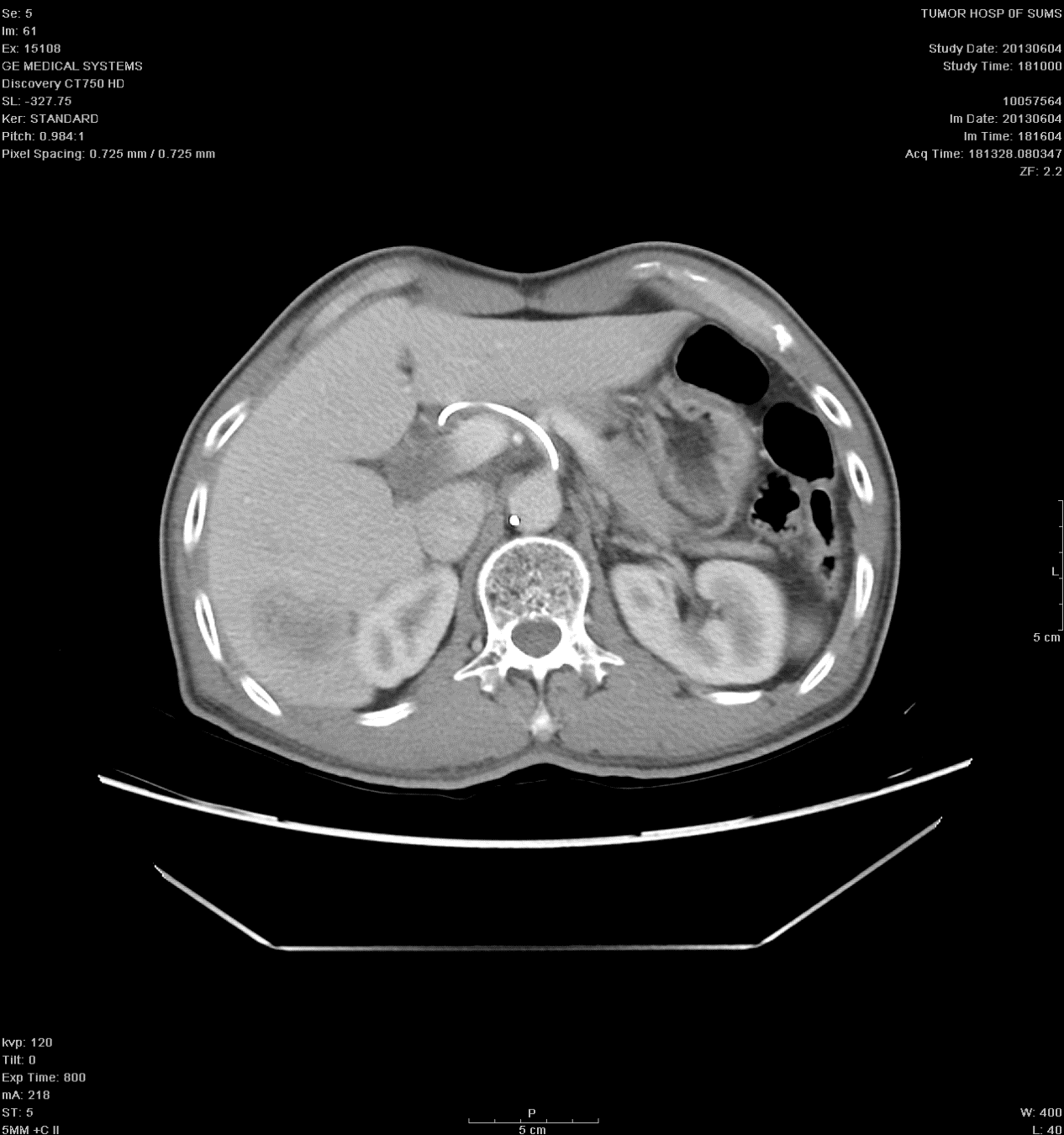


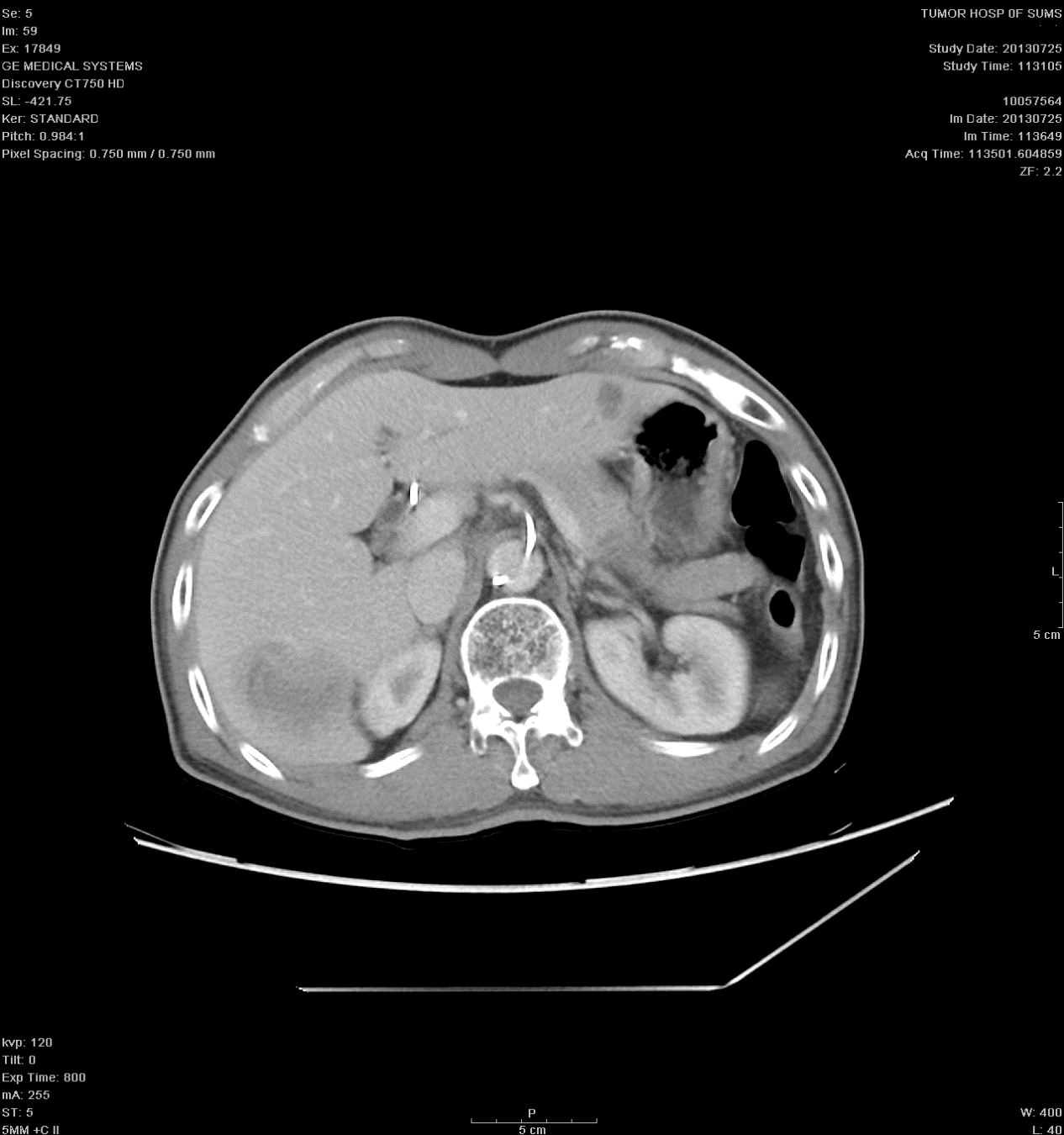


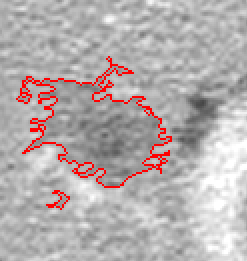

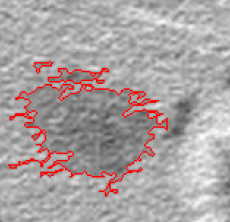

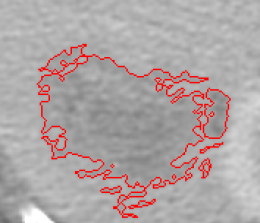

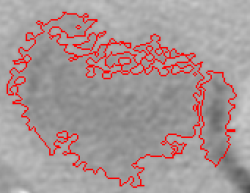


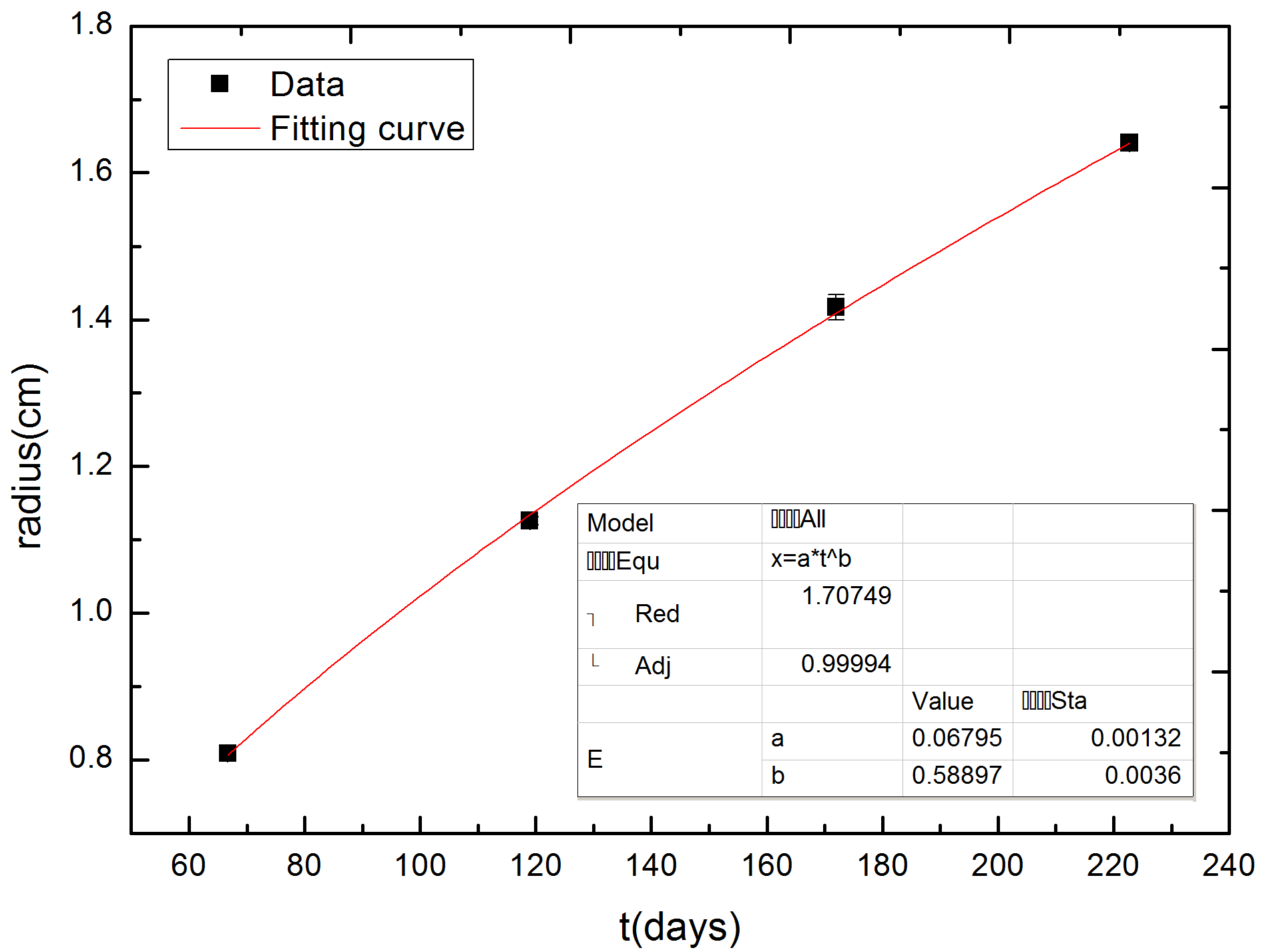


Patient3: Liver tumors B, C and D


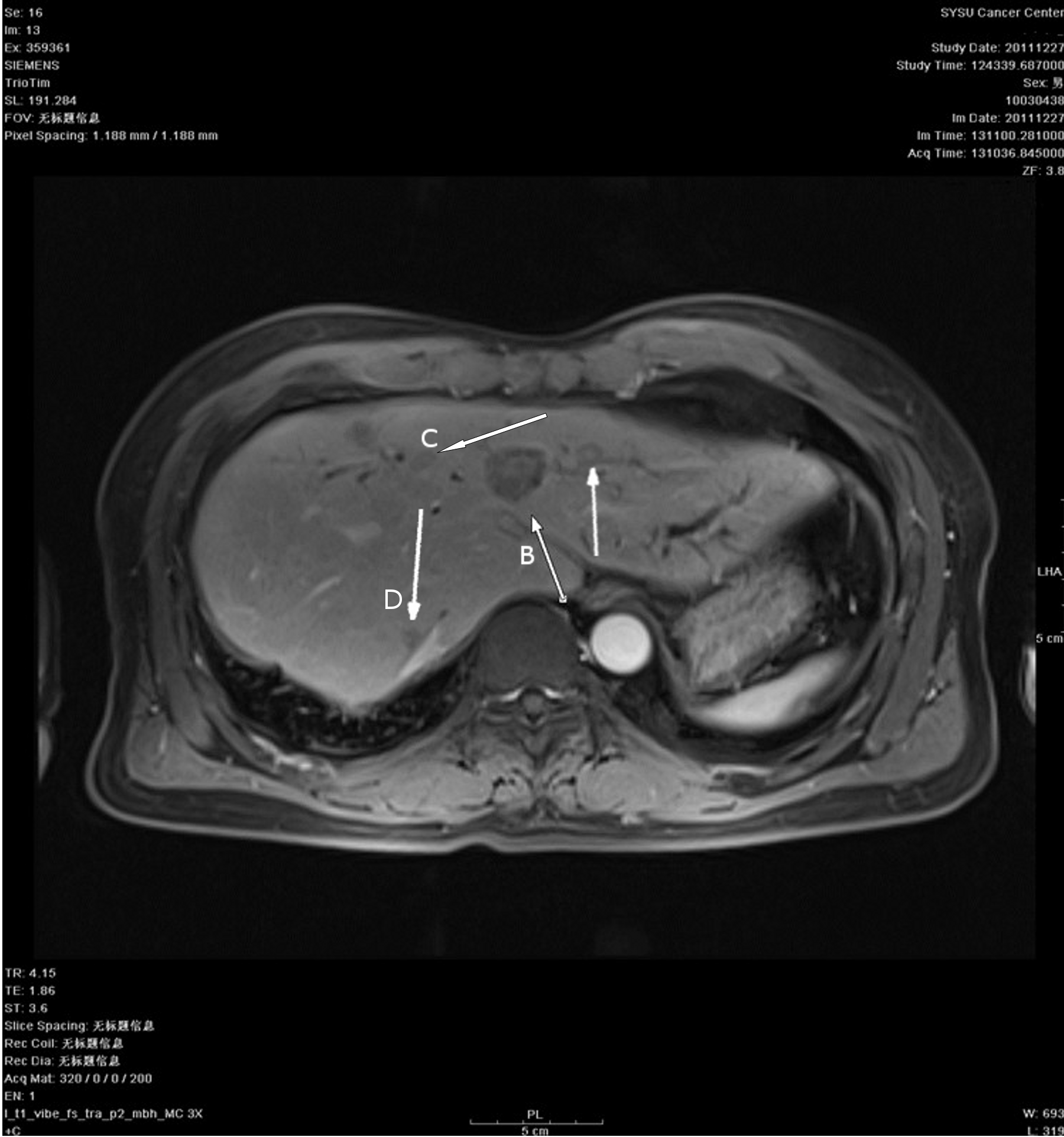


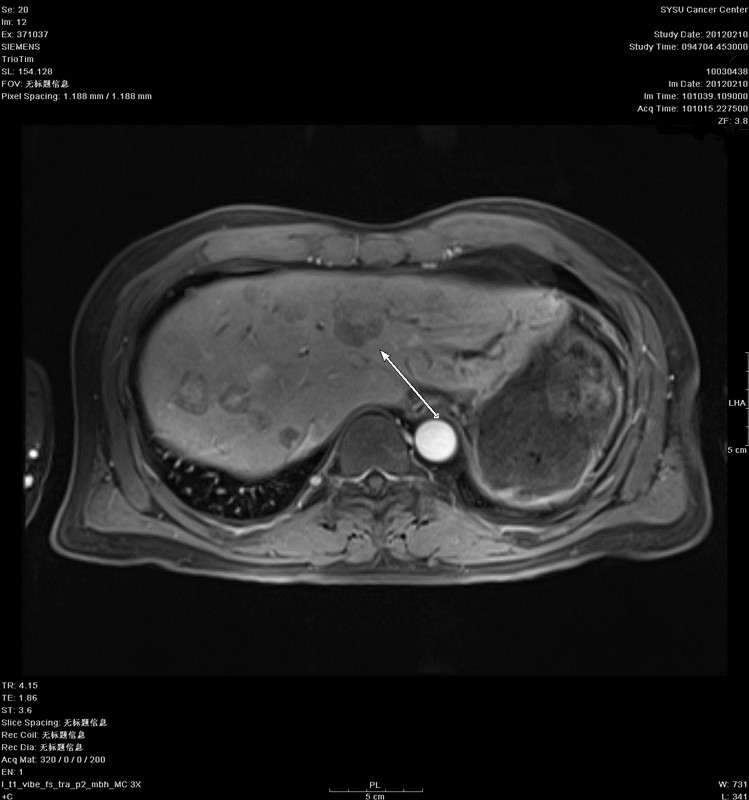


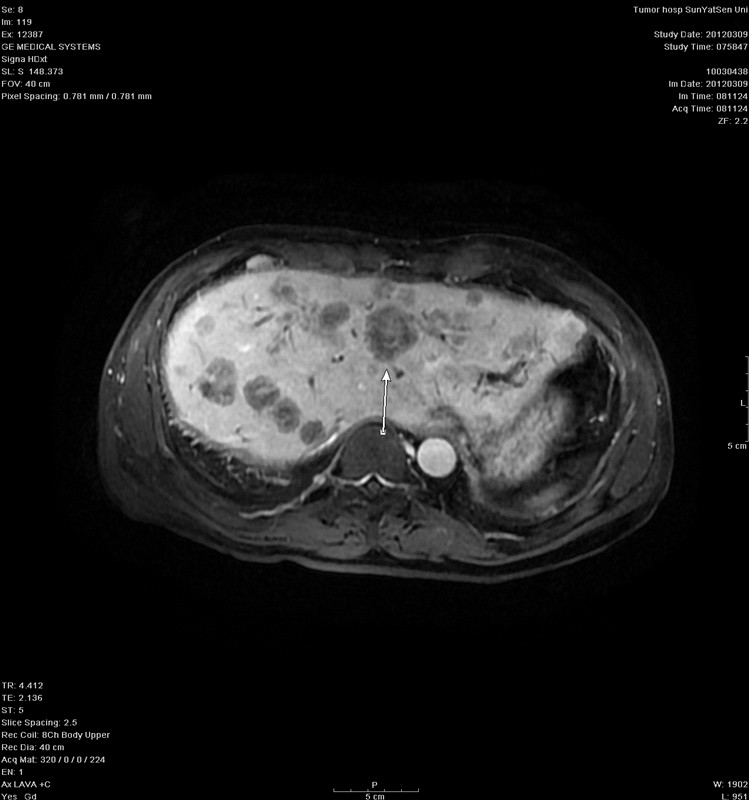


Liver tumors B


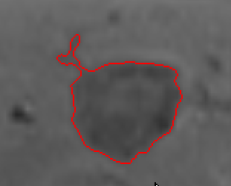

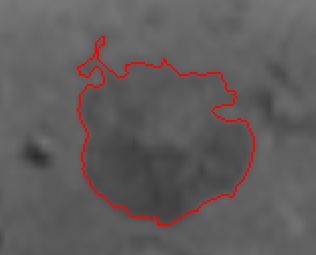

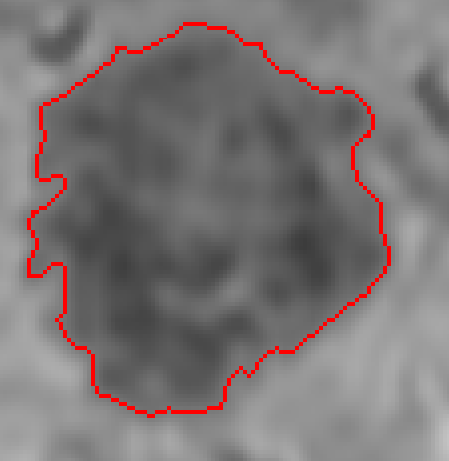


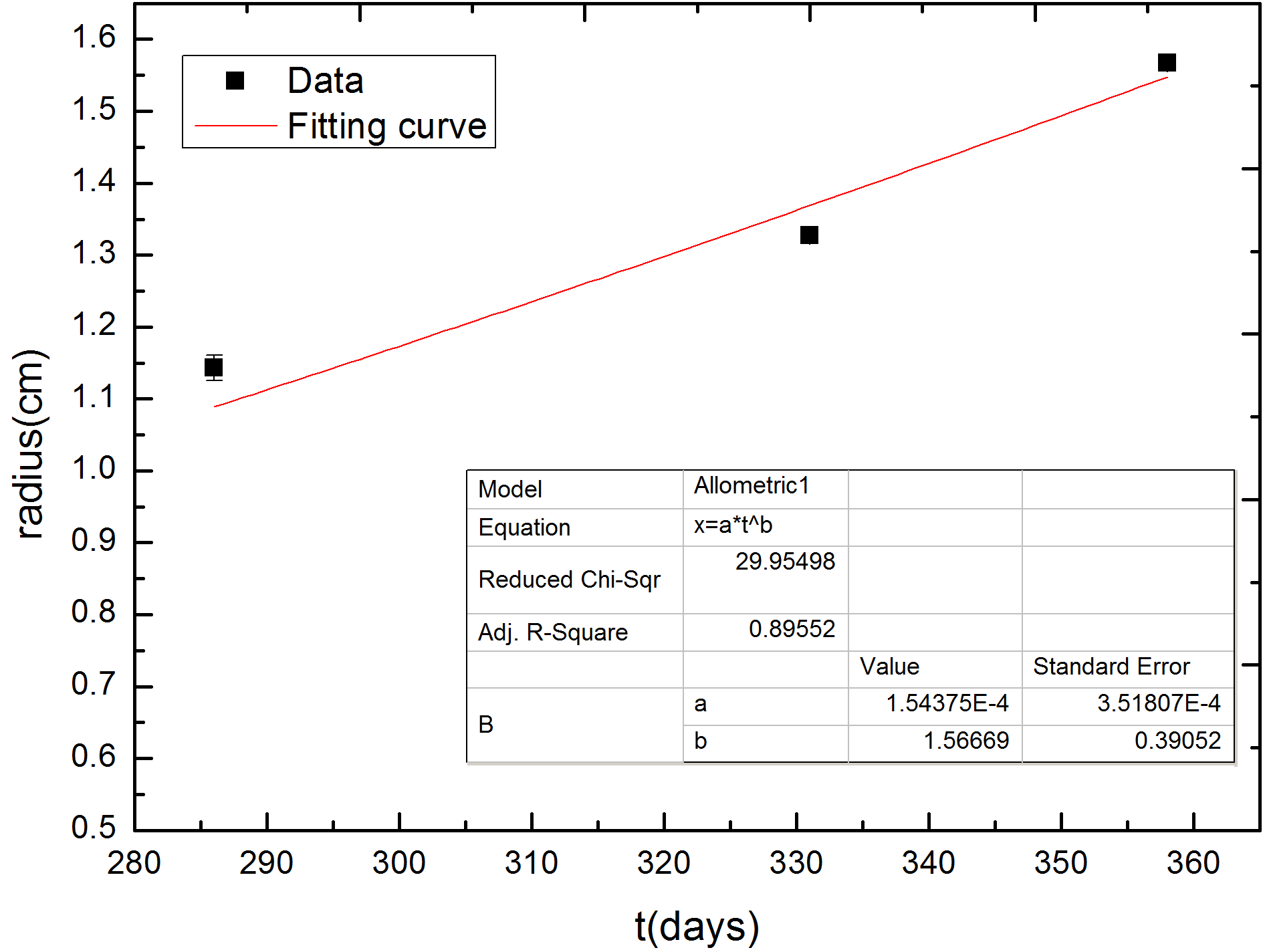


Liver tumors C


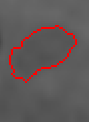

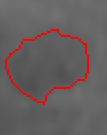

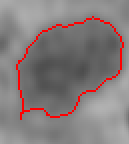


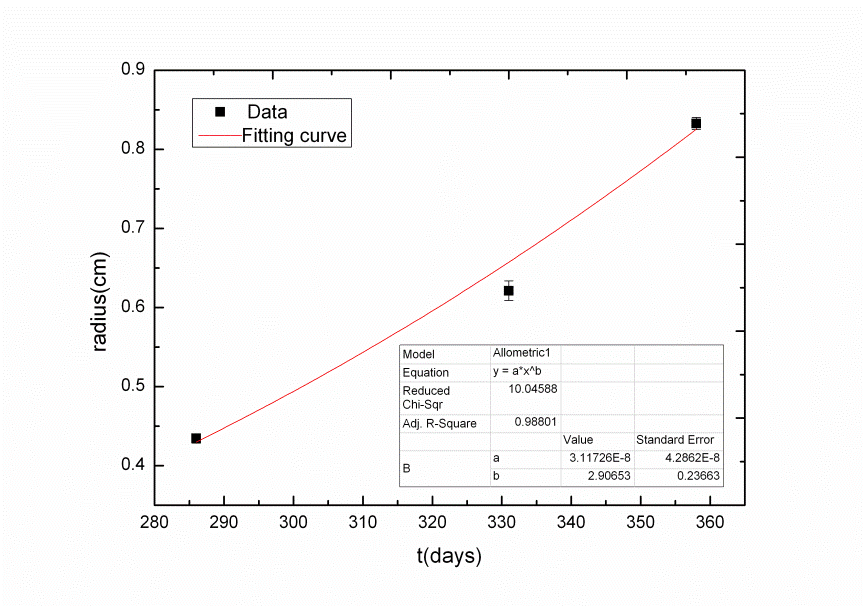


Liver tumors D


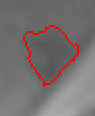

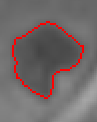

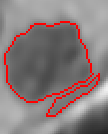


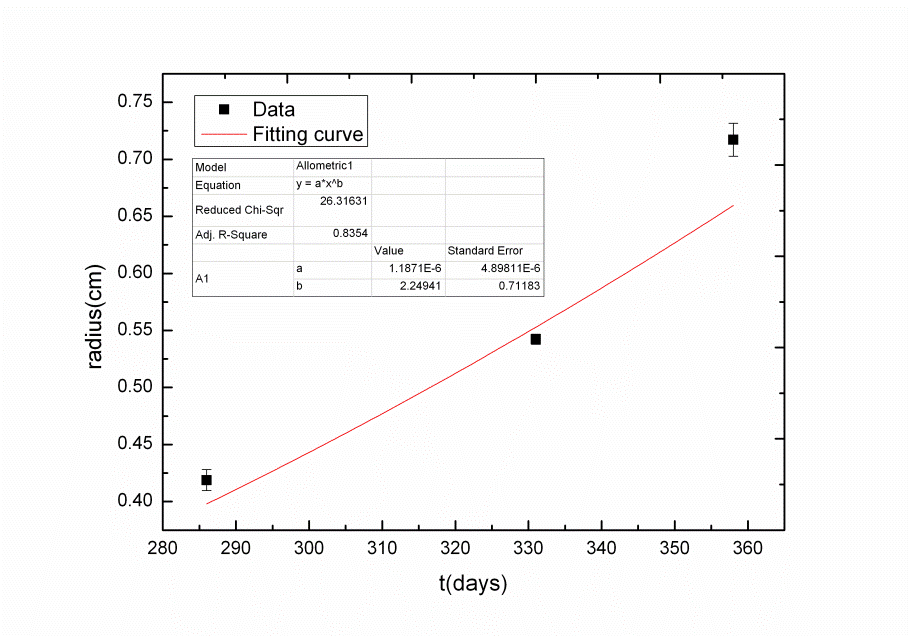

Supplement: File S4 — The original graphs and curve fitting of Clinical images. The Sun Yat-sen University Cancer Center provided data from three cancer patients who had been clinically diagnosed with a metastatic adrenal tumor (patient 1 with an expansive growing tumor) or a metastatic liver tumor (patient 2 with an infiltrative growing tumor, patient 3 with three infiltrative growing tumors). The liver tumor lesions in the two patients were numbered tumors A, B, C, and D (liver tumor A is the liver metastasis from choroidal melanoma with low-grade malignancy; liver tumor B, C, and D are the liver metastases from colorectal cancer with high-grade malignancy). This study was approved by the ethics committee of Sun Yat-sen University Cancer Center and protected the patients' private information. (DOC) [file pone.0109784.s005.doc]
